# Deletion of *Cryab* increases the vulnerability of mice to the addiction-like effects of the cannabinoid JWH-018 *via* upregulation of striatal NF-κB expression

**DOI:** 10.3389/fphar.2023.1135929

**Published:** 2023-03-16

**Authors:** Leandro Val Sayson, Darlene Mae Ortiz, Hyun Jun Lee, Mikyung Kim, Raly James Perez Custodio, Jaesuk Yun, Chae Hyeon Lee, Yong Sup Lee, Hye Jin Cha, Jae Hoon Cheong, Hee Jin Kim

**Affiliations:** ^1^ Department of Pharmacy, Uimyung Research Institute for Neuroscience, Sahmyook University, Seoul, Republic of Korea; ^2^ Department of Chemistry and Life Science, Sahmyook University, Seoul, Republic of Korea; ^3^ Department of Ergonomics, Leibniz Research Centre for Working Environment and Human Factors—IfADo, Dortmund, Germany; ^4^ College of Pharmacy and Medical Research Center, Chungbuk National University, Cheongju, Chungcheongbuk-do, Republic of Korea; ^5^ Medicinal Chemistry Laboratory, Department of Fundamental Pharmaceutical Sciences, College of Pharmacy, Kyung Hee University, Seoul, Republic of Korea; ^6^ College of Veterinary Medicine, Gyeongsang National University, Jinju, Gyeongsangnam–do, Republic of Korea; ^7^ Institute for New Drug Development, School of Pharmacy, Jeonbuk National University, Jeonju, Jeollabuk-do, Republic of Korea

**Keywords:** cannabinoid, *Cryab*, JWH-018, neuroinflammation, NF-κB, synaptic plasticity, addiction susceptibility, drug abuse

## Abstract

Synthetic cannabinoids have exhibited unpredictable abuse liabilities, especially self-administration (SA) responses in normal rodent models, despite seemingly inducing addiction-like effects in humans. Thus, an efficient pre-clinical model must be developed to determine cannabinoid abuse potential in animals and describe the mechanism that may mediate cannabinoid sensitivity. The *Cryab* knockout (KO) mice were recently discovered to be potentially sensitive to the addictive effects of psychoactive drugs. Herein, we examined the responses of *Cryab* KO mice to JWH-018 using SA, conditioned place preference, and electroencephalography. Additionally, the effects of repeated JWH-018 exposure on endocannabinoid- and dopamine-related genes in various addiction-associated brain regions were examined, along with protein expressions involving neuroinflammation and synaptic plasticity. *Cryab* KO mice exhibited greater cannabinoid-induced SA responses and place preference, along with divergent gamma wave alterations, compared to wild-type (WT) mice, implying their higher sensitivity to cannabinoids. Endocannabinoid- or dopamine-related mRNA expressions and accumbal dopamine concentrations after repeated JWH-018 exposure were not significantly different between the WT and *Cryab* KO mice. Further analyses revealed that repeated JWH-018 administration led to possibly greater neuroinflammation in *Cryab* KO mice, which may arise from upregulated NF-κB, accompanied by higher expressions of synaptic plasticity markers, which might have contributed to the development of cannabinoid addiction-related behavior in *Cryab* KO mice. These findings signify that increased neuroinflammation *via* NF-κB may mediate the enhanced addiction-like responses of *Cryab* KO mice to cannabinoids. Altogether, *Cryab* KO mice may be a potential model for cannabinoid abuse susceptibility.

## 1 Introduction

The development of synthetic cannabinoids was initially aimed at understanding the endocannabinoid system and developing potential pharmacotherapies for cannabinoid-induced disorders (de Luca and Fattore, 2018; Diao and Huestis, 2019). However, the growing number of novel synthetic cannabinoids has become a major public health concern as the identification of specific synthetic cannabinoids consumed by abusers became difficult. Additionally, novel cannabinoids are classified under the highest drug schedule due to their adverse effects and lack of medical use (Drug Enforcement Administration, 2015). Prevalence of their abuse might be due to their stronger effects compared to trans-Δ⁹-tetrahydrocannabinol (THC), the main psychoactive component of marijuana (Weinstein et al., 2017), and being “legally” available online. Despite inducing adverse effects (MacDonald and Pappas, 2016; Schmitz and Richert, 2020; National Institute on Drug Abuse, 2021), synthetic cannabinoids have persistently circumvented legal regulations and initial detection owing to their varying chemical composition, resulting from the constant modification of functional groups. Thus, evaluating the potential dangers of these substances has piqued the interest of the scientific community.

The pharmacological properties of synthetic cannabinoids are similar to those of exogenous phytocannabinoids, with synthetic cannabinoids being more potent due to their higher affinity for cannabinoid receptors (Tai and Fantegrossi, 2014). Recent studies have suggested that CB_1_ receptors are ubiquitously expressed throughout the central nervous system, forming part of the endocannabinoid system (Fagundo et al., 2013), which is interconnected with the mesolimbic dopaminergic pathway, a signaling network implicated in mediating drug addiction (Adinoff, 2004; Vlachou and Panagis, 2014). The endocannabinoid system may mediate addiction-like behaviors by regulating *γ*-Aminobutyric acid (GABA)/glutamate neurotransmission *via* retrograde signaling of CB_1_ receptors expressed on GABAergic/glutamatergic neurons, thus influencing dopaminergic neuronal activity (Parsons and Hurd, 2015). This may provide the basis for cannabinoid-induced addiction in humans, leading to cannabinoid use disorders. However, some studies have demonstrated that not all synthetic cannabinoids can induce significant addiction-like behaviors in animal models (Polissidis et al., 2009; Hyatt and Fantegrossi, 2014; Tampus et al., 2015; Rodríguez-Arias et al., 2016; Bilel et al., 2019), especially in self-administration (SA) paradigms, contradictory to clinical reports and personal accounts (Le Boisselier et al., 2017; de Luca and Fattore, 2018; Leung et al., 2020). This presents several challenges in screening the abuse potential of novel cannabinoids and the involvement of complex neural mechanisms in the pathophysiology of cannabinoid abuse. Thus, there is a need to develop an efficient tool for evaluating the abuse potential of synthetic cannabinoids.

Accumulating evidence suggest the involvement of neuroinflammation in cannabinoid addiction, potentially entailing both pro- and anti-inflammatory activities (Kozela et al., 2010; Kinsey et al., 2011; Javed et al., 2016; Bayazit et al., 2017). Some cannabinoids also seem to mitigate the addictive effects of abused drugs (Xi et al., 2011; Delis et al., 2017; Lin et al., 2018). Increased neuroinflammation induced by drugs of abuse may modulate glutamate synapses *via* increased astroglia glutamate reuptake (Boycott et al., 2008; Ramos et al., 2010), thus affecting glutamate-dependent synaptic plasticity (Park et al., 2015), which may reinforce drug-seeking behavior (Kauer and Malenka, 2007). Since some cannabinoids may reduce cytokine production (Xu et al., 2007) and deactivate glial cells (Rodrigues et al., 2014), their potential effects on neuroinflammation may provide another possible mechanism that may also mediate cannabinoid addiction development. This further suggests their inconsistent addiction-inducing effects in rodent models to probably involve inflammatory processes.

Transgenic mice models have been widely used to elucidate the pathophysiology of substance abuse disorder (Spanagel and Sanchis-Segura, 2003; Sora et al., 2010). These models are commonly developed based on genes that are differentially expressed in response to drugs of abuse. These genes are then modified in mice to generate transgenic rodent models (Yazdani et al., 2015; Szumlinski et al., 2017). Preliminary findings have suggested that treatment with methamphetamine or cocaine modulates the expression of *Cryab* in mice (Ministry of Food and Drug Safety, 2021). *Cryab* codes a small heat shock protein (CRYAB) with anti-inflammatory functions, such as reducing apoptosis through enhancing PI3K and activating AKT signaling (Ren et al., 2018), and inhibiting inflammatory responses in glial cells by downregulating pro-inflammatory mediators (Kuipers et al., 2017; Guo et al., 2019). Although initially, CRYAB functions as a molecular chaperone and aggregates misfolded proteins (Dai et al., 2022), thus preventing detrimental protein accumulation during stress (Calderwood et al., 2019). Furthermore, it may also participate in various types of cancer (Zhang et al., 2019), providing it a potential anti-inflammatory and anti-apoptotic role. Although the administration of addictive drugs may alter the expression of *Cryab*, the role of *Cryab* in cannabinoid-induced addiction has not yet been investigated. Therefore, a *Cryab* transgenic mouse model may be possibly used to determine the abuse potential of novel substances and identify susceptibility to potentially addictive drugs, such as synthetic cannabinoids.

Thus, this study aimed to provide an animal model that would be susceptible to cannabinoid abuse by determining the responses of *Cryab* knockout (KO) mice to 1-naphthalenyl(1-pentyl-1H-indol-3-yl)-methanone (JWH-018; a representative cannabinoid) in addiction-associated behavioral paradigms. Given the effect of *Cryab* on inflammation and the involvement of inflammation in addiction development, *Cryab* KO mice might exhibit increased sensitivity to JWH-018 abuse. As neural electrical activity indicates physiological responses to drugs of abuse, the electroencephalogram of *Cryab* KO mice may perhaps also differ from those of wild-type (WT) mice after repeated JWH-018 exposure. Since cannabinoid addiction may also involve endocannabinoid, dopaminergic, and neuroinflammatory pathways, repeated exposure to JWH-018 might differentially modulate endocannabinoid-related, dopamine-related, and neuroinflammation-related genes and/or proteins in *Cryab* KO mice, contributing to their divergent cannabinoid-induced behaviors when compared with WT mice.

## 2 Materials and methods

### 2.1 Animals

**FIGURE 1:**
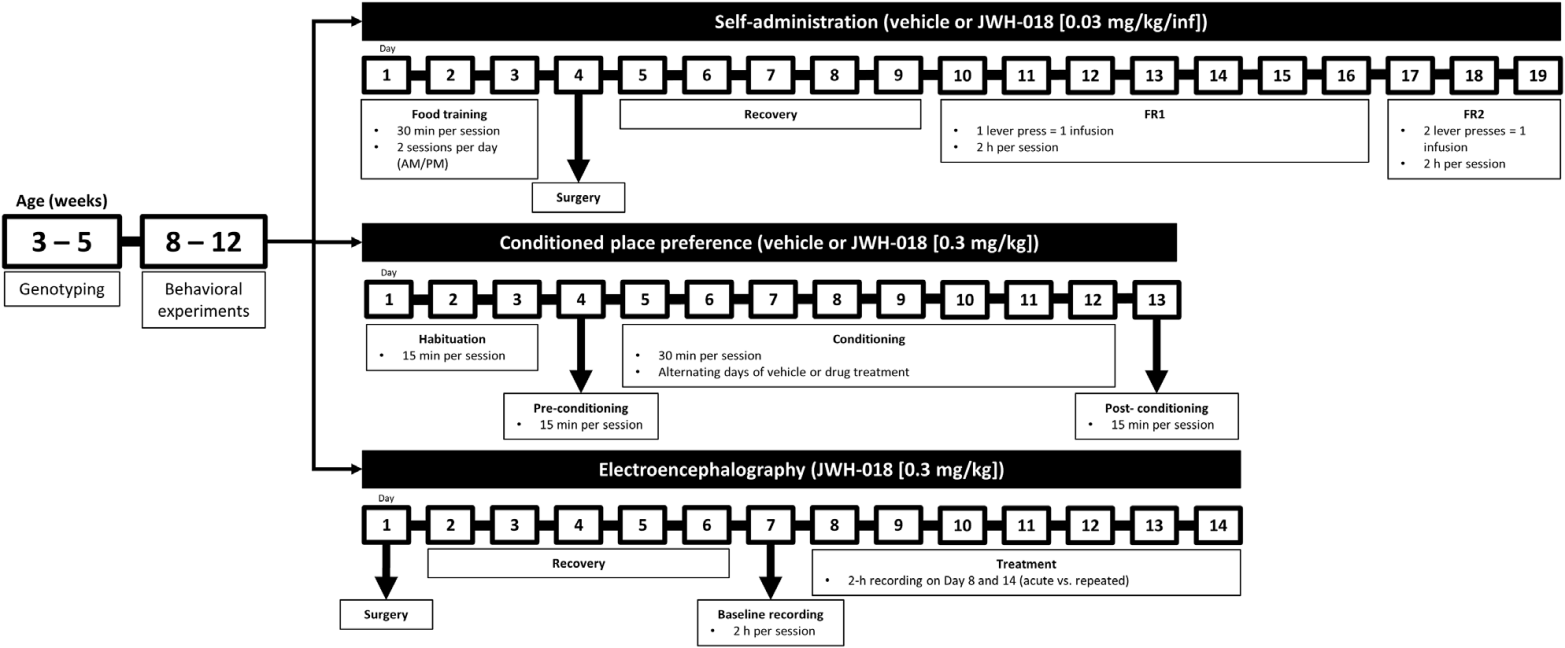
Schedule of behavioral experiments. Mice are genotyped between 3 and 5 weeks of age using tail samples. Mice are then segregated according to genotype. Starting at 8 weeks of age, only male mice are housed according to the experiment. Brains may be collected at the end of each behavioral experiment.

The Department of Pharmacy, Chungbuk National University (Cheongju City, Korea) provided two male and two female *Cryab* KO mice (aged 8 weeks). Transgenic mice were bred with C57BL/6N mice (aged 8 weeks, 22–25 g) obtained from Hanlim Animal Laboratory Co. (Hwasung, Korea), to obtain heterozygous *Cryab* (*Cryab* Het) mice. All procured mice were acclimatized in the animal room for 1 week prior to being used. Male and female *Cryab* Het mice (aged 8 weeks, F1) were subsequently bred to obtain male *Cryab* KO mice (F2), which were used for all experiments in this study (aged 8–12 weeks, 25–30 g). Newborn pups were genotyped at 3–4 weeks old using DNA from the tail. Only male mice were used for behavioral and molecular experiments. Mice for experiments were housed together in cages (4–6 mice per cage). To obtain next generation mice (F3), male and female *Cryab* Het mice (F2) were bred. All mice were housed in a room under controlled conditions (circadian cycle, 12-h light/dark cycle (7 AM–7 PM); temperature, 22°C ± 2°C) and had access to food *ad libitum*. The schedule of experiments is presented in Figure 1. Animal treatment and maintenance procedures were performed according to the Principles of Laboratory Animal Care (NIH Publication No. 85–23, revised 1985) and the Animal Care and Use Guidelines of Sahmyook University, South Korea (SYUIACUC 2021-020 and SYUIACUC 2022-001).

### 2.2 Drugs

JWH-018 was synthesized according to reported procedures (Huffman et al., 2005). In brief, indole was reacted with pentyl bromide and potassium hydroxide in DMF. The obtained *N*-pentylindole was acylated with 1-naphthoyl chloride in the presence of Me_2_AlCl in dichloromethane to produce JWH-018. The structure was confirmed by the following spectroscopic analyses: ^1^H-NMR (500 MHz, CDCl_3_) δ 8.52-8.48 (m, 1H), 8.20 (d, *J* = 8.6 Hz, 1H), 7.95 (t, *J* = 8.3 Hz, 1H), 7.90 (d, *J* = 8.0 Hz, 1H), 7.65 (dd, *J* = 6.9, 1.1 Hz, 1H), 7.54-7.45 (m, 3H), 7.40-7.35 (m, 4H), 4.05 (t, *J* = 7.4 Hz, 2H), 1.82-1.76 (m, 2H), 1.32-1.22 (m, 4H), 0.85 (t, *J* = 7.2 Hz, 3H); ^13^C-NMR (125 MHz, CDCl_3_) δ 192.1, 139.2, 138.1, 137.1, 133.8, 130.9, 130.1, 128.3, 127.1, 126.8, 126.4, 126.1, 125.9, 124.7, 123.7, 123.0, 122.9, 117.6, 110.1, 47.3, 29.6, 29.0, 22.3, 14.0; HR-MS calculated for C_24_H_23_NO [M + H]^+^ 342.1852, found 342.1852; HPLC purity 99.46%. In all experiments, JWH-018 was dissolved in a solution comprising Tween 80, absolute ethanol, and 0.9% (w/v) saline (SAL) in the ratio 1:1:48 to obtain the desired dosage (0.3 mg/kg body weight). JWH-018 was selected as it has been used in several early cannabinoid-based studies, and the dose was selected based on reports that demonstrated significant addiction-related effects of JWH-018 and other cannabinoids in rodents (de Luca et al., 2015; Ossato et al., 2017).

### 2.3 Behavioral tests

#### 2.3.1 Open-field test (OFT)

Baseline locomotor activity was evaluated using a square Plexiglas open-field arena (42 cm × 42 cm × 42 cm), as per the methodology described in previous studies (Custodio et al., 2018; dela Peña et al., 2019; Kim et al., 2019). Mice were placed at the center of the arena and allowed to explore for 12 min for three consecutive days. The recorded data of the first 2 min were excluded from the analysis, as this period was considered a habituation period. The distance moved (cm) and the movement duration (s) of each mouse was measured using an automated system (EthoVision, Noldus, Netherlands).

#### 2.3.2 Y-maze test

Basic cognition in mice was assessed using a “Y”-shaped maze comprising three identical arms (each arm spaced at an angle of 120°) with each arm measuring 5 cm × 35 cm × 10 cm. Each mouse was placed at the end of an arm and allowed to explore the maze freely for 8 min. Spontaneous alternating behavior and total entry in each arm were obtained following a previous study (Custodio et al., 2018). The alternation behavior (actual alternation) was described as the consecutive entry into three arms (continuous exploration of three different arms (e.g., ABC, BCA, or CAB) with stepwise sequence combinations. The maximum number of alternations was defined as the total number of arms entered minus two. The percentage spontaneous alternation behavior was calculated as follows: spontaneous alternation behavior (%) = (actual alternations/maximum alternations) × 100.

#### 2.3.3 Rota-rod test

General motor balance and coordination of mice were measured using a rotating rod (Ugo Basile, Varese, Italy) at a fixed speed of 36 rpm, as per the methodology described in previous studies (Custodio et al., 2018; dela Peña et al., 2019). Two consecutive days before the experiment, the mice were allowed to habituate and were trained to run on the rotating rod for 3 min (T1 and T2). On the day of the experiment, “challenge day” (Ch), the mice were placed on the rotating rod for 10 min. Mice that fell during the experiment were gently placed back onto the rota-rod. The falling latency and frequency were recorded.

#### 2.3.4 Cliff avoidance test

The impulsive behavior of the mice was assessed using a 50 cm long cylindrical Plexiglass rod support with a round Plexiglass platform (diameter = 20 cm; thickness = 1 cm) on both ends. Each mouse was placed at the center of the platform, and its behavior was observed for 10 min. The first fall latency and falling frequency were recorded.

#### 2.3.5 Elevated plus-maze test (EPM)

Inherent anxiety-like behavior in mice was determined using a plus-maze comprising four arms, two open and two closed arms measuring 30 cm × 6 cm (closed arms were enclosed by 20 cm high walls). The center of the maze contained a 6 cm × 6 cm delimited area. At the start of each test, individual mice were placed at the center, facing one of the open arms. The percentage of entries (100 × open/total entries) and time spent in the open arms were calculated after each 5 min test, similar to a previous study (dela Peña et al., 2019). The EthoVision system was used for automated recording and analysis.

#### 2.3.6 Electroencephalography (EEG)

The mice were anesthetized with 0.02 mL Zoletil^®^ (50 mg/mL) and Rompun^®^ (xylazine 23.32 mg/mL) prepared in SAL. A three-channel tethered head mount (8200K3-iS/iSE) was placed and fixed with stainless screws positioned in the frontal region (A/P: −1.0 mm, M/L −1.5 mm) and posterior brain (A/P: −1.0 mm, M/L ±1.5 mm), conductive epoxy, and dental cement. Standard operating procedures were performed to minimize animal discomfort. The mice were allowed to recover for 5 days before recording. Data acquisition and analyses were performed according to the methodology described in previous studies (Botanas et al., 2021; Custodio et al., 2021) but with some modifications. On the last recovery day, mice were allowed to acclimatize to the EEG apparatus for 2 h (unrecorded). On the next day, a baseline recording for drug-naïve mice was performed for 2 h with an initial 10 min of habituation using a computer-based software (Pinnacle Technology, Inc., Lawrence, KS, United States). One group of mice was treated with JWH-018 (0.3 mg/kg body weight) or vehicle (VEH) for 7 days. After the initial baseline recording (Day 0), EEG was recorded on the first and last day of treatment. EEG wave changes relative to the baseline recording were calculated.

#### 2.3.7 Intravenous self-administration (SA) test

The SA test was performed as reported previously (Custodio et al., 2022). Lever pressing training, which was based on food pellet reward under continuous reinforcement, was performed for three consecutive days [30 min per session (AM and PM sessions)]. In this study, lever pressing behavior was determined when mice earned 15 reinforcers at the end of the session on the third day and 75% of responses were on the active lever (Thomsen and Caine, 2011). After training, surgery was performed to insert a 0.28 mm inner diameter catheter into the jugular vein and mid-scapular region of mice anesthetized with Zoletil^®^ (50 mg/mL) and Rompun^®^ (xylazine 23.32 mg/mL) prepared in SAL. The catheterized mice were allowed to recover for 5 days. During recovery, the catheters were infused daily with 0.02 mL of 0.9% SAL containing heparin (20 IU/mL) and gentamicin (0.08 mg/mL). Catheter patency was determined by flushing catheters with a 0.02 mL of the Zoletil^®^/Rompun^®^ mixture, and the loss of muscle tone was observed in mice within 10 s of infusion. During the SA experiment, catheters were flushed with SAL containing gentamicin or heparin immediately before and after each SA session. Mice were assessed daily in 2-h SA sessions under a fixed-ratio (FR) 1 schedule for 7 days, followed by assessment with an FR2 schedule for 3 days (Graphic State Notation 4; Coulbourn Instruments, Whitehall, PA, United States). Mice were fed a pellet diet (2 g) daily. In each session, both left and right levers were available. Left (active) lever pressing was followed by infusion of 0.05 mL JWH-018 (0.03 mg/kg body weight/infusion) or SAL over 10 s. During the infusion, the stimulus light over the lever was illuminated for 20 s. Lever presses during “time-out” periods were recorded but had no effect. Right (inactive) lever presses were also recorded, but not reinforced. Active and inactive lever presses and the number of infusions for each daily session were recorded for evaluating SA behavior.

#### 2.3.8 Conditioned place preference (CPP) test

The apparatus comprised two compartments (dimensions = 17.4 cm × 12.7 cm × 12.7 cm) with a removable guillotine door that separated the compartments. One compartment had smooth black walls and white flooring, whereas the other had white-dotted black walls and textured white flooring. An illumination of 12 lux was maintained throughout the experiment. A computer system (EthoVision) was used for recording animal movements and stay durations in the compartments. The protocol was performed according to previous studies with some modifications (Kim et al., 2019; Custodio et al., 2020; Sayson et al., 2020). The test comprised the following three phases: (A) habituation (days 1–3) and pre-conditioning (day 4; 15 min), (B) conditioning (days 5–12; 30 min), and (C) post-conditioning (day 13; 15 min). During habituation, mice were allowed to freely explore the entire apparatus. An initial trial (pre-conditioning) was performed to determine the stay duration of each mouse in each of the compartments. Mice were assigned to groups based on the pre-conditioning phase such that their non-preferred side was designated as the drug-paired compartment. In the conditioning phase, mice were administered JWH-018 (0.3 mg/kg body weight) or VEH and placed in the drug-paired compartment. On alternate days, the mice received VEH and were confined to the VEH-paired compartment. During the post-conditioning phase, the mice were not treated and were allowed to explore both compartments (similar to the pre-conditioning phase). The CPP score was calculated as the difference in the time spent by the mice in their respective drug-paired compartments between the post-conditioning and pre-conditioning phases.

### 2.4 Molecular experiments

#### 2.4.1 RNA extraction and quantitative real-time polymerase chain reaction (qRT-PCR)

Drug-naïve or JWH-018-treated mice (7 days) were euthanized and decapitated to excise the brain 30 min after the last treatment. The ventral tegmental area (VTA) and nucleus accumbens (NAC) were isolated using a mouse brain matrix on ice. The samples were rapidly frozen at −80°C before further processing. The subsequent steps followed previous methods (Custodio et al., 2022) and were according to MIQE guidelines. Total RNA was isolated with TRIzol^®^ (Invitrogen, Carlsbad, CA, United States), following the manufacturer’s protocol, and purified using the Hybrid-RTM kit (Geneall Biotechnology, Seoul, Korea). RNA concentrations were determined using Colibri Microvolume Spectrometer (Titertek-Berthold, Pforzheim, Germany). Total RNA (1 μg) was reverse-transcribed into complementary DNA (cDNA) using AccuPower^®^ CycleScript RT PreMix (Bioneer, Seoul, Korea), following the manufacturer’s instructions. Aliquots of cDNA were stored at −20°C. Target genes were amplified using custom sequence-specific primers (Cosmogenetech, Seoul, Korea) and detected using SYBR^®^ Green (Solgent, Daejeon, Korea). The primer sequences are shown in Supplementary Table S1. The input cDNA concentration was 2.5 μg/μL. The PCR conditions were as follows: 94°C for 1 min (denaturing step), followed by annealing at primer-specific temperature for 1 min, and 72°C for 45 s. qRT-PCR was performed using samples in triplicate. The expression of target genes was normalized to that of *Gapdh*. The results are expressed as relative expression calculated using the 2^−ΔΔCT^ method (VanGuilder et al., 2008).

#### 2.4.2 Protein extraction and enzyme-linked immunosorbent assay (ELISA)

Brains of a separate cohort of VEH or JWH-018-exposed (7-day treatment) mice were collected and isolated similarly as described in the previous section. Protein extraction was done according to previous methods with slight modifications (Sayson et al., 2020). Brain tissues were lysed in 400 μL homogenization buffer [radioimmunoprecipitation assay buffer (Biosesang Inc., Seongnam, Korea) supplemented with cOmplete™ ULTRA protease inhibitor cocktail tablets (05892791001, Sigma-Aldrich) and PhosSTOP™ phosphatase inhibitor cocktail tablets (04906845001, Sigma-Aldrich)]. The tissue extracts were centrifuged at 16,000 *g* at 4°C for 20 min. Dopamine concentration was determined using the dopamine ELISA kit (KA1887, Abnova, Taiwan), following the manufacturer’s instructions. Briefly, standards, controls, and samples were acylated and incubated with dopamine antiserum for 2 h at room temperature (20°C–25°C) on a shaker (approximately 600 rpm). Next, the samples in the wells were washed and incubated with the enzyme conjugate for 30 min. After washing the samples, the substrate was pipetted into individual wells, and the samples were incubated for 25 min. The stop solution was then added to each well. The absorbance of the reaction mixture was measured at 450 nm using an E_Max_ Plus Microplate Reader (Molecular Devices; San Jose, CA, United States). All measurements were performed in duplicates.

#### 2.4.3 Western blotting

The striatum (STR) of VEH or JWH-018-exposed (7-day treatment) mice was isolated. Procedure was done according to previous studies (Sayson et al., 2020; Custodio et al., 2021). The samples were then heated at 95°C for 5 min. Protein lysates (20 μg) were subjected to sodium dodecyl sulfate-polyacrylamide gel electrophoresis on a 12% gel. The resolved proteins were transferred onto nitrocellulose membranes. The membrane was blocked with 5% bovine serum albumin (BSA) prepared in Tris-buffered saline containing 0.1% Tween-20 (TBST) for 1 h and then incubated overnight with specific primary antibodies at 4°C. Then, the membrane was washed with TBST and incubated with horseradish peroxidase-conjugated anti-rabbit (1:3,000) or anti-mouse secondary antibodies (1:5,000) for 1 h. Protein bands were visualized based on enhanced chemiluminescence (Clarity Western ECL; Bio-Rad Laboratories, Hercules, CA, USA) using the ChemiDoc Imaging System (Image Lab software, version 6.0; Bio-Rad). The levels of phosphorylation-independent proteins were normalized to those of *β*-Actin. The levels of the phosphorylated form of proteins were normalized to those of the total form of proteins. Fold change was determined by normalizing the values of the test groups to those of the WT VEH group. The antibodies used for the Western blot analysis are listed in Supplementary Table S2.

#### 2.4.4 Immunofluorescence

Standard protocols were used, following previously described methods (Custodio et al., 2022). After the last SA session, the mice were intracardially perfused with perfusion solution [0.05 M phosphate-buffered saline (PBS)] and perfusate [4% paraformaldehyde (PFA) in 0.1 M phosphate buffer]. The brain tissues were stored in PFA solution at 4°C overnight, followed by incubation with 30% sucrose solution at 4°C. The brain samples were sectioned to a thickness of 35 μm using a Leica CM1850 cryostat (Wetzlar, Germany), following mouse brain stereotaxic coordinates (Paxinos and Franklin, 2001). For this experiment, the STR was selected for analysis as this region is commonly implicated in inflammation-associated behavioral alterations, such as addiction (Krasnova et al., 2016; Vonder Haar et al., 2019). The brain sections were carefully washed with 1× PBS and subsequently incubated in a protein-blocking solution (5% BSA and 0.3% Triton X-100 in 1× PBS) for 1 h at room temperature. Next, the sections were incubated with primary antibodies diluted in a protein-blocking solution for 3 days at 4°C. The primary antibody used in this analysis is listed in Supplementary Table S2. After washing, the samples were incubated overnight with Alexa fluor-555-conjugated goat anti-mouse (Thermo Fisher Scientific A32727, RRID: AB_2633276) antibody at 4°C. The samples were washed, incubated with Hoechst for 10 min, and mounted on 76 mm × 26 mm × 1 mm clean positively charged microscope slides (Walter Products Inc., Canada). Mounted sections were cured with Permount^®^ Mounting Medium UN1294 (Fisher Chemical, NJ, United States), covered with 24 mm × 50 mm microscope cover glasses (Marienfeld Laboratory Glassware, Germany), and allowed to dry for 24 h at room temperature. The corrected total cell fluorescence (CTCF) level was measured in each sample using ImageJ 1.53k (NIH, Maryland, United States) as reported previously (Hammond, 2014).

### 2.5 Statistical analyses

All mice were randomized for treatment. The researchers were blinded to the treatment of the animals while conducting the tests and analyzing the data. Statistical analyses were performed using GraphPad Prism v7 (GraphPad Software Inc., San Diego, CA, United States). Data are presented as the mean ± standard error of the mean (S.E.M.), unless specified otherwise. The means were analyzed using one-way or two-way analysis of variance (ANOVA) with or without repeated measures (RM), followed by Tukey’s or Bonferroni’s multiple comparison test, when appropriate. Differences were considered statistically significant at *p* < 0.05. The *p*-value and detailed statistical analysis (e.g., genotype, treatment, time, days, etc.) are indicated.

## 3 Results

### 3.1 *Cryab* KO mice exhibited hypoactivity

**FIGURE 2:**
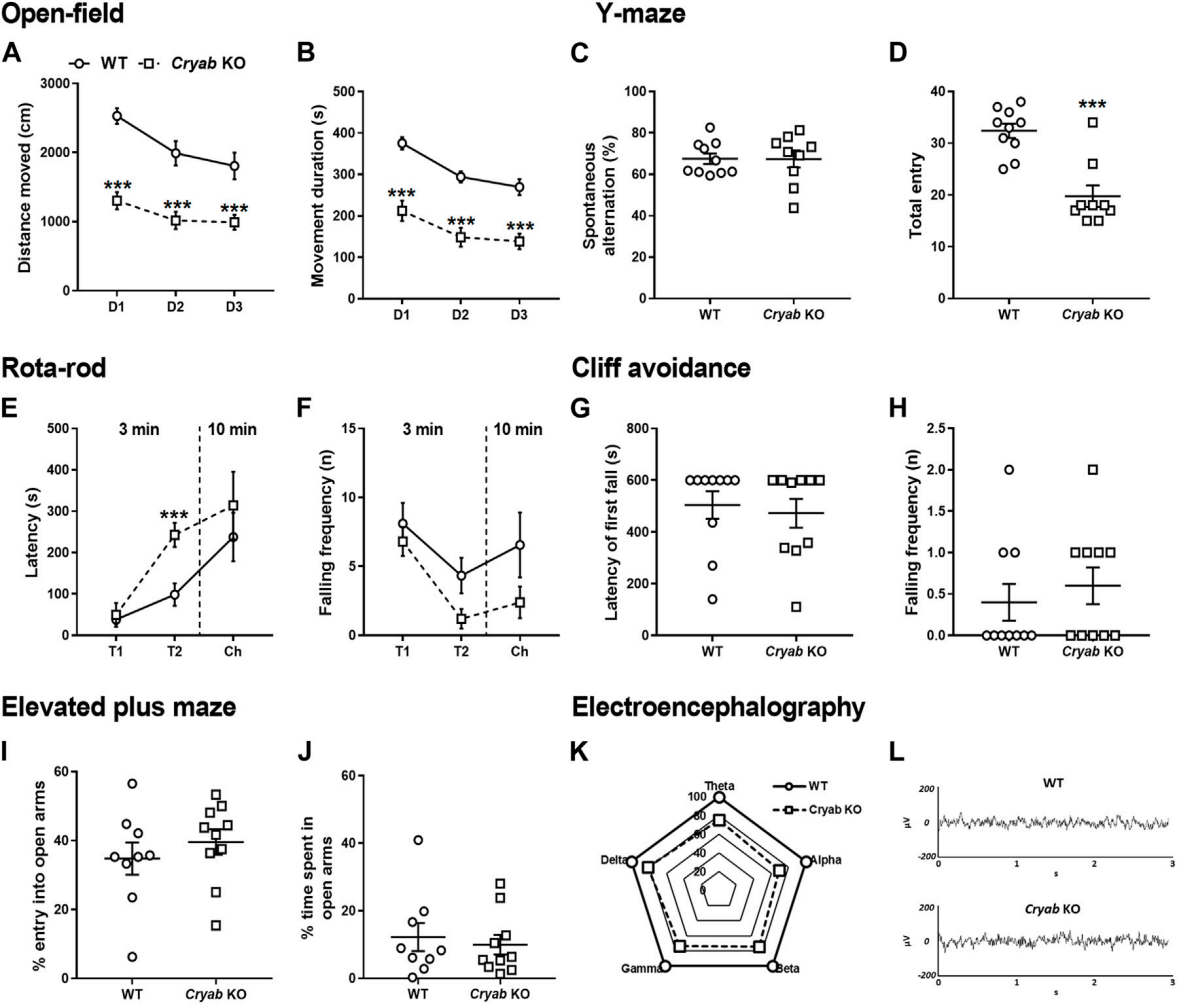
General behavior of wild-type (WT) and *Cryab* knockout (KO) mice. At 5–7 weeks old, pups were tested for their baseline behaviors. The open-field test shows the locomotor activity indicated by **(A)** distance moved (cm) and **(B)** movement duration (s) recorded for 10 min over three consecutive days. Y-maze indicates short-term memory in terms of **(C)** spontaneous alternating behavior (%) and **(D)** total entries. Latency of first fall (s) and falling frequency (n) in the **(E,F)** rota-rod and **(G,H)** cliff avoidance tests determine motor balance/coordination and impulsive tendencies, respectively. Anxiety is measured by the **(I)** percentage entry (%) and **(J)** percentage time spent (%) in the open arms in the elevated maze plus test. **(K)** Baseline electroencephalogram (EEG) results reveals the power of delta, theta, alpha, beta, and gamma in mice, recorded for 2 h (drug-free). **(L)** Sample EEG traces shows a 2-s activity during a 2-h recording. Data expressed as mean ± S.E.M., except **(K)** and **(L)**. *n* = 9–10. ***p < 0.001 (vs. WT; Bonferroni *post hoc* analysis or *t*-test).

Although controversial, inherent aberrant behaviors were previously correlated with substance abuse co-morbidity (Herrero et al., 2008; Volkow, 2009; Kabir et al., 2016), therefore we screened the general behavior of *Cryab* KO mice in various behavioral assays. OFT revealed that *Cryab* KO mice innately possessed lower spontaneous locomotor activity than WT mice, as evidenced by their lower total distance moved (Figure 2A; genotype: F_1,18_ = 29.6; *p* < 0.001; test day: F_2,36_ = 27.0; *p* < 0.001) and movement duration (Figure 2B; genotype: F_1,18_ = 37.3; *p* < 0.001; test day: F_2,36_ = 35.1; *p* < 0.001), even across multiple testing sessions. Spontaneous alternation (Figure 2C) in the Y-maze were similar between mice genotypes, while total entry (Figure 2D; *t* = 5.14, df = 17) also suggested lower locomotor activity in *Cryab* KO mice. Other behaviors in rota-rod (Figures 2E, F), cliff avoidance (Figures 2G, H), and EPM (Figures 2I, J) exhibited no differences between WT and *Cryab* KO mice. Drugs of abuse also modulate neural electrical activity (Newton et al., 2003; Malyshevskaya et al., 2017; Zanettini et al., 2019; Nukitram et al., 2021), suggesting that EEG alterations may co-manifest alongside the addiction-inducing effects of some psychoactive drugs. Aberrant EEG patterns may also indicate potentially modified neuropsychological responses to addictive drugs. Accordingly, 2-h baseline EEG recording data of mice (Figure 2K) showed that the delta-, theta-, alpha-, beta-, and gamma-wave levels of *Cryab* KO mice were lower than WT mice [absolute power (μV^2^) of WT mice was set to 100%]. Sample EEG traces of mice (Figure 2L) showed comparable patterns between genotype.

### 3.2 *Cryab* KO mice exhibited increased responses in addiction-related behavioral assays and divergent gamma-wave power

**FIGURE 3:**
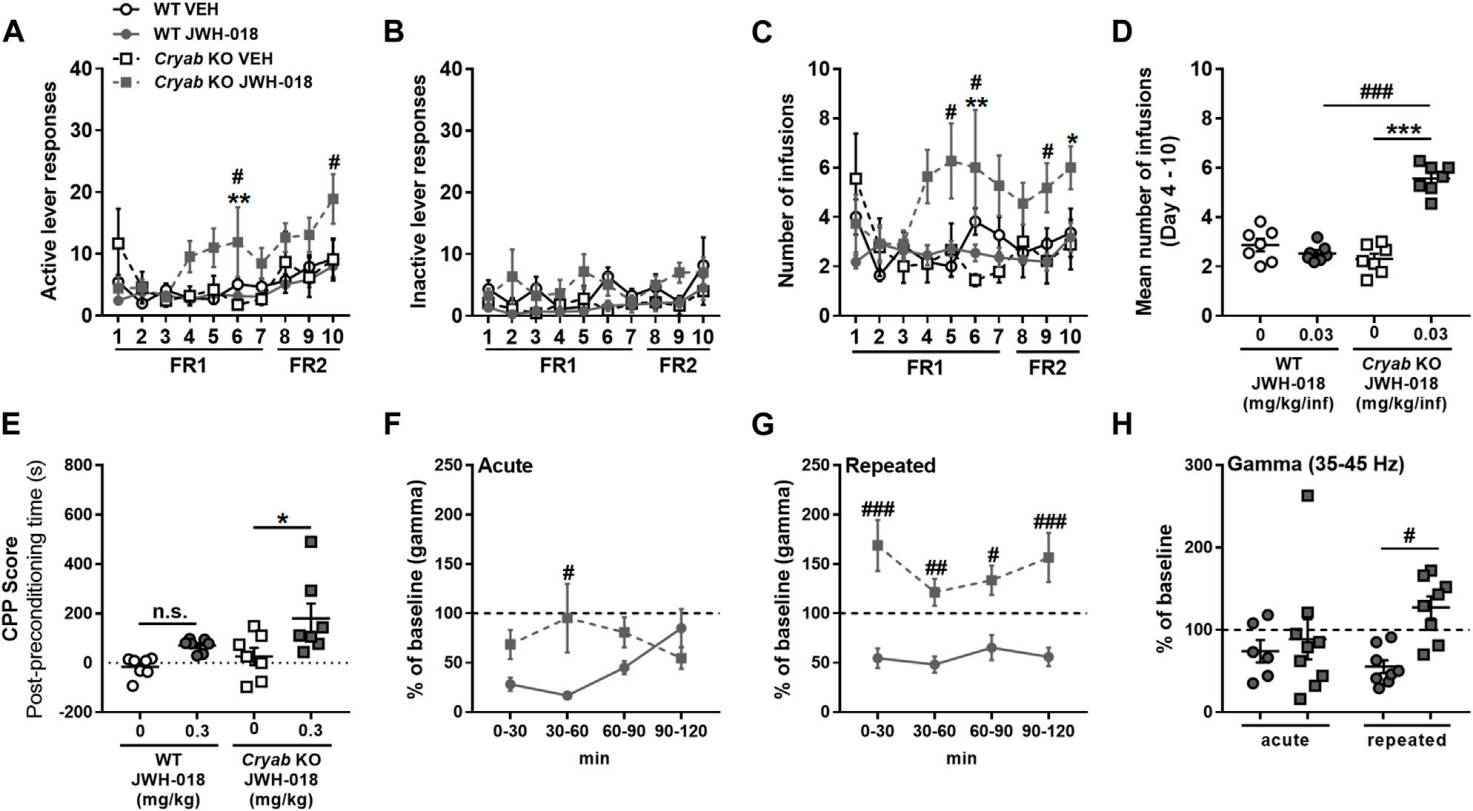
Response of wild-type (WT) and *Cryab* knockout (KO) mice to JWH-018 addiction-related behavioral paradigms. Self-administration (SA) responses of mice are indicated by **(A)** active and **(B)** inactive lever responses and the **(C)** number of infusions, along with **(D)** mean number of infusions, which were recorded each day for a 2-h session. *n* = 9–11. Place preference is indicated by **(E)** Conditioned place preference (CPP) score of mice, which is obtained by the difference between the stay duration of mice in the drug-paired compartment between post- and pre-conditioning. *n* = 7. Changes in gamma-wave power of mice after **(F)** acute or **(G)** repeated treatments over 30-min bins were determined by 2-h electroencephalography (EEG) recordings, and the 2-h averages **(H)** were compared. *n* = 6–9. Data expressed as mean ± S.E.M. *p < 0.05, **p < 0.01, and ***p < 0.001 (vs. VEH; Tukey *post hoc* analysis). ^#^ *p* < 0.05, ^##^ *p* < 0.01, and ^###^ *p* < 0.001 (vs. WT; Tukey *post hoc* analysis).

To evaluate the responses of mice to the reinforcing effect of JWH-018, we exposed WT and *Cryab* KO mice to JWH-018 SA. *Cryab* KO obtained higher active lever responses (Figure 3A) in FR1 (F_3,38_ = 3.94; *p* < 0.05) and FR2 [treatment (F_3,38_ = 2.99; *p* < 0.05); SA days (F_2,76_ = 4.38; *p* < 0.05)], along with greater number of infusions (Figure 3C) and FR1 (F_3,38_ = 4.9; *p* < 0.01) and FR2 (F_3,38_ = 3.49; *p* < 0.05), compared to WT mice. Average obtained infusions (Figure 3D) all throughout the SA sessions were also significantly higher (F_3,24_ = 51.3; *p* < 0.001) in *Cryab* KO mice compared to their WT and VEH counterparts. The varying behavior of mice during SA indicate the manifestation of JWH-018-induced reinforcing effects in *Cryab* KO mice. Aside from that, the rewarding property of JWH-018 was also evaluated in WT and *Cryab* KO mice through the CPP paradigm. JWH-018 appeared to induce rewarding effects in *Cryab* KO mice (Figure 3E), as evidenced by their higher CPP score (F_3,24_ = 5.59; *p* < 0.01) compared to their VEH counterpart, but not in WT mice.

Gamma-wave generation is also altered in chronic cannabis users owing to their continuous intake of cannabinoids (Skosnik et al., 2012; Liu et al., 2022), as evidenced by *in vivo* and *in vitro* studies (Sales-Carbonell et al., 2013). Thus, gamma wave alterations in mice following repeated JWH-018 administration were also determined through EEG. Gamma-wave power after acute JWH-018 treatment (Figure 3F) was significantly different among mice with a treatment-time interaction (F_3,42_ = 5.71; *p* < 0.01), specifically the change in gamma-wave power in *Cryab* KO mice was lesser than WT mice only after 60 min post-treatment. Repeated JWH-018 administration also induced significant differences in change in gamma-wave power (Figure 3G) among mice (F_3,42_ = 5.71; *p* < 0.01), showing that gamma-wave power in *Cryab* KO mice increased significantly from baseline, unlike WT mice, all throughout the recording period. The change in gamma-wave power in mice during the 2-h recording (Figure 3H) was significantly different among groups (F_3,27_ = 3.21; *p* < 0.05). The change in gamma-wave power of *Cryab* KO mice was significantly higher than WT mice after repeated JWH-018 treatment, but not after acute exposure.

### 3.3 Expressions of endocannabinoid system-related and dopamine-related genes, including accumbal dopamine concentration, were not markedly different between genotypes

**FIGURE 4:**
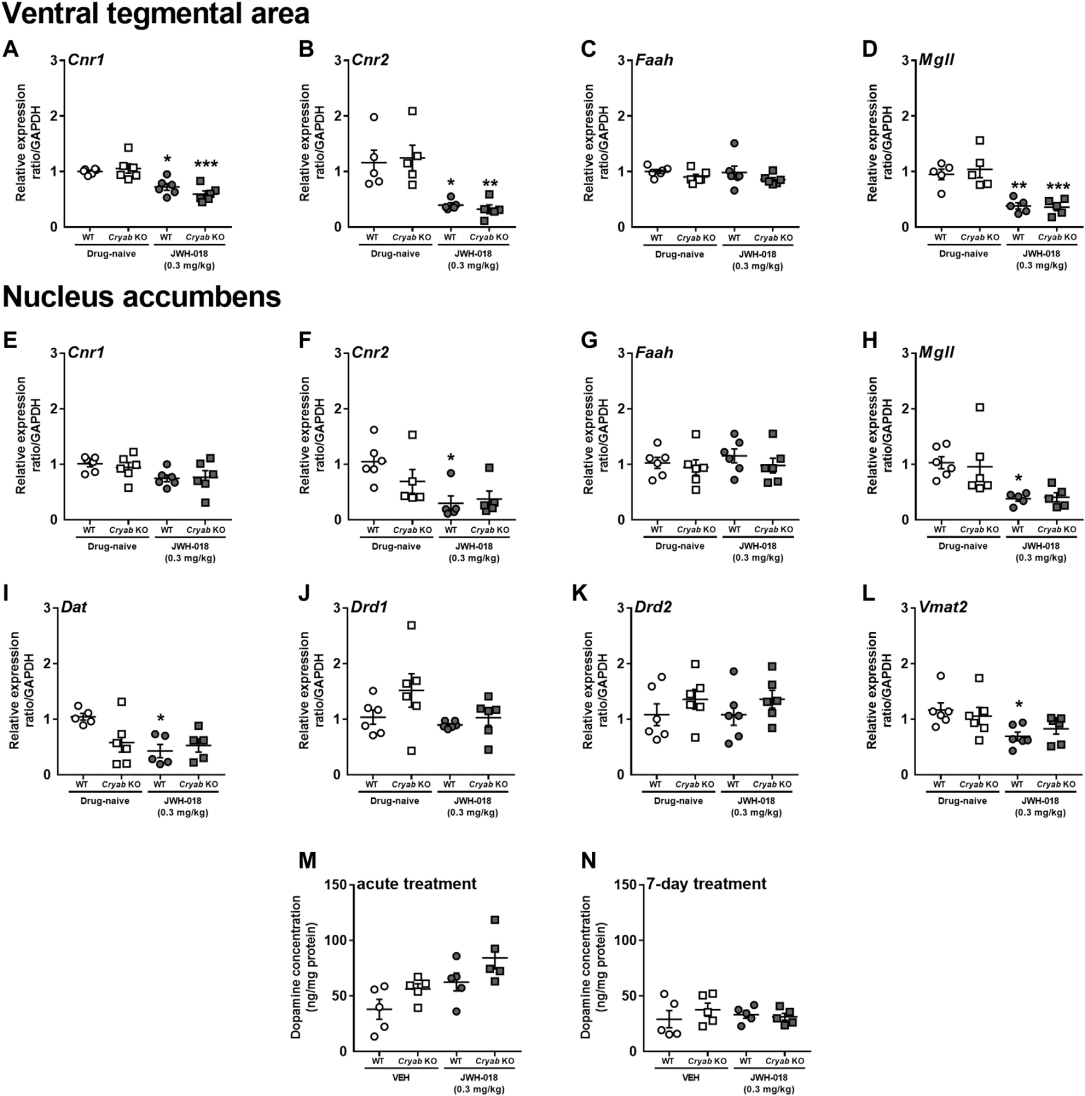
Expression levels of endocannabinoid system-related and dopamine-related genes and dopamine concentration in wild-type (WT) and *Cryab* knockout (KO) mice after repeated JWH-018 treatment. Expressions of *Cnr1*, *Cnr2*, *Faah*, and *Mgll* in the **(A–D)** ventral tegmental area (VTA) and **(E–H)** nucleus accumbens (NAC) were determined through quantitative real-time polymerase chain reaction (qRT-PCR) following repeated JWH-018 treatment. The expression levels of **(I)** *Dat*, **(J)** *Drd1*, **(K)** *Drd2*, and **(L)** *Vmat2* in the NAC of mice following repeated JWH-018 treatment were also identified through qRT-PCR. Enzyme-linked immunosorbent assay determined the dopamine concentrations in mouse NAC after **(M)** acute or **(N)** repeated JWH-018 treatment. Data expressed as mean ± S.E.M. *n* = 4–6. *p < 0.05, **p < 0.01, ***p < 0.001 (vs. drug-naïve; Tukey *post hoc* analysis).

The abuse liabilities of THC and synthetic cannabinoids are usually attributed to their influence on CB_1_ receptors expressed in the VTA and NAC, which are major brain regions associated with the development of addiction-related behaviors (Nestler, 2001). Cannabinoids may also affect the expression of CB_2_ receptors (Sun et al., 2017), along with enzymes that degrade endocannabinoids (Li et al., 2019). Thus, we measured the mRNA levels of endocannabinoid-related genes in the VTA and NAC of mice repeatedly treated with JWH-018. *Cnr1* (Figure 4A; F_1,20_ = 38.8; *p* < 0.001), *Cnr2* (Figure 4B; F_1,16_ = 26.2; *p* < 0.001), and *Mgll* (Figure 4D; F_1,16_ = 41; *p* < 0.001) mRNA levels were downregulated in the VTA of WT and *Cryab* KO mice following repeated JWH-018 treatment. However, *Cnr1*, *Cnr2*, *Faah*, and *Mgll* mRNA levels in the VTA were not significantly different between WT and *Cryab* KO mice. In the NAC, *Cnr2* (Figure 4F; F_1,17_ = 10.9; *p* < 0.01) and *Mgll* (Figure 4H; F_1,18_ = 16.4; *p* < 0.001) mRNA levels were also downregulated only in WT mice following repeated JWH-018 exposure. Similar to the VTA, *Cnr1*, *Cnr2*, *Faah*, and *Mgll* mRNA levels in the NAC were also not significantly different between genotypes. *Faah* expressions in both VTA and NAC (Figures 4C, G) were unaltered, although *Cnr1* expression in NAC (Figure 4E) exhibited treatment differences between groups (F_1,20_ = 6.65; *p* < 0.05).

Since endocannabinoid signaling within the VTA may influence dopaminergic neurotransmission, divergent addiction responses in mice genotypes might be reflected by differential cannabinoid-mediated regulation of the VTA and distinct changes in dopamine-related markers in the NAC. Accordingly, we determined the mRNA levels of dopamine-related genes in the NAC of mice following repeated JWH-018 treatment. No significant alterations were detected in *Drd1* (Figure 4J) and *Drd2* (Figure 4K) mRNA levels, whereas the expression levels of *Dat* (Figure 4I; F_1,16_ = 8.23; *p* < 0.05) and *Vmat2* (Figure 4L; F_1,20_ = 8.63; *p* < 0.01) in WT mice were downregulated. We also determined the accumbal dopamine concentration in mice following acute and repeated JWH-018 administration to verify the potential effect of cannabinoid treatment on dopamine-mediated responses. Accumbal dopamine concentrations in mice showed no significant differences between the genotypes following repeated JWH-018 treatment (Figure 4N), although significant differences (treatment: F_1,16_ = 10.4; *p* < 0.01; genotype: F_1,16_ = 6.04; *p* < 0.05) were observed among groups following acute JWH-018 treatment (Figure 4M)*.*

### 3.4 *Cryab* KO mice demonstrated potentially higher neuroinflammatory state after JWH-018 exposure

**FIGURE 5:**
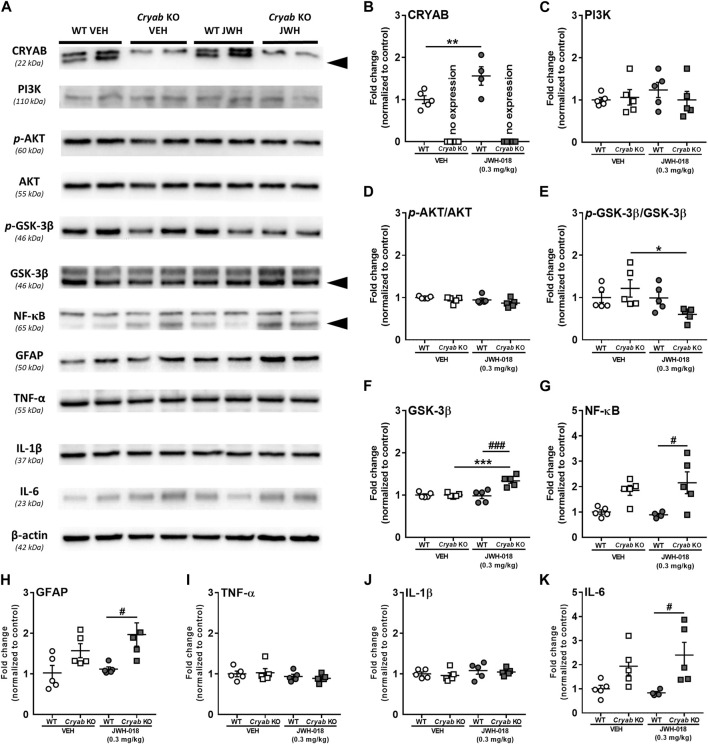
Expression of Protein expressions along the PI3K-AKT-GSK3 pathway and inflammation-related proteins in wild-type (WT) and *Cryab* knockout (KO) mice following JWH-018 exposure. **(A)** Representative blots showing the expression of target proteins in mice STR. Western blotting revealed the expression levels of **(B)** CRYAB, **(C)** PI3K, **(D)** *p*-AKT, **(E)** GSK-3β, **(F)** *p*-GSK-3β, **(G)** NF-κB, **(H)** GFAP, **(I)** TNF-α, **(J)** IL-1β, and **(K)** IL-6 in the STR of JWH-018-treated mice. *n* = 4–5. Data expressed as mean ± S.E.M. *p < 0.05, **p < 0.01, and ***p < 0.001 (vs. VEH; Tukey’s *post hoc* analysis). ^#^ *p* < 0.05 and ^###^ *p* < 0.001 (vs. WT; Tukey’s *post hoc* analysis).

Striatal neuroinflammatory processes may possibly potentiate addiction-related behavior (Rodrigues et al., 2014; Kohno et al., 2019). Studies have mentioned that CRYAB may produce anti-inflammatory effects by inhibiting cytokine expression through various pathways (Shao et al., 2013; Qiu et al., 2016; Somade et al., 2019; Xu et al., 2019). Thus, CRYAB may influence addiction development through its impact on neuroinflammation. In here, we first identified the effect of CRYAB expression on the inflammation-related PI3K-AKT-GSK3 pathway (Xu et al., 2013), which may also modulate NF-κB expression (Xie and Wang, 2022), following repeated JWH-018 administration. Representative blots are shown in Figure 5A. JWH-018 exposure increased CRYAB expression (Figure 5B; genotype: F_1,15_ = 156; *p* < 0.001; treatment: F_1,15_ = 7.51; *p* < 0.05) in WT mice. PI3K (Figure 5C) and *p*-AKT levels (Figure 5D) were not significantly different between groups. *p*-GSK-3β levels (Figure 5E; treatment: F_1,16_ = 4.85; *p* < 0.05) were decreased in *Cryab* KO mice following repeated JWH-018 administration, while GSK-3β expression (Figure 5F; genotype: F_1,16_ = 15; *p* < 0.01, treatment: F_1,16_ = 11.5; *p* < 0.01) was significantly higher in *Cryab* KO mice than their WT and VEH counterpart. Interestingly, repeated JWH-018 administration in the absence of functional CRYAB resulted in a higher NF-κB expression (Figure 5G) in *Cryab* KO mice than WT mice (F_1,15_ = 17.2; *p* < 0.001).

NF-κB may also influence neuroinflammation by mediating inflammatory cytokine gene transcription (Liu et al., 2017). Accordingly, we verified the expression of glial cell activity and neuroinflammatory markers in mice STR. While TNF-α (Figure 5I) and IL-1β (Figure 5J) were unaltered in mice after JWH-018 exposure, GFAP (Figure 5H; F_1,16_ = 13.1; *p* < 0.001) and IL-6 (Figure 5K; F_1,15_ = 12.7; *p* < 0.01) expressions were higher in *Cryab* KO mice than WT mice.

### 3.5 *Cryab* KO mice exhibited potentially higher glutamate-dependent synaptic plasticity after JWH-018 exposure

**FIGURE 6:**
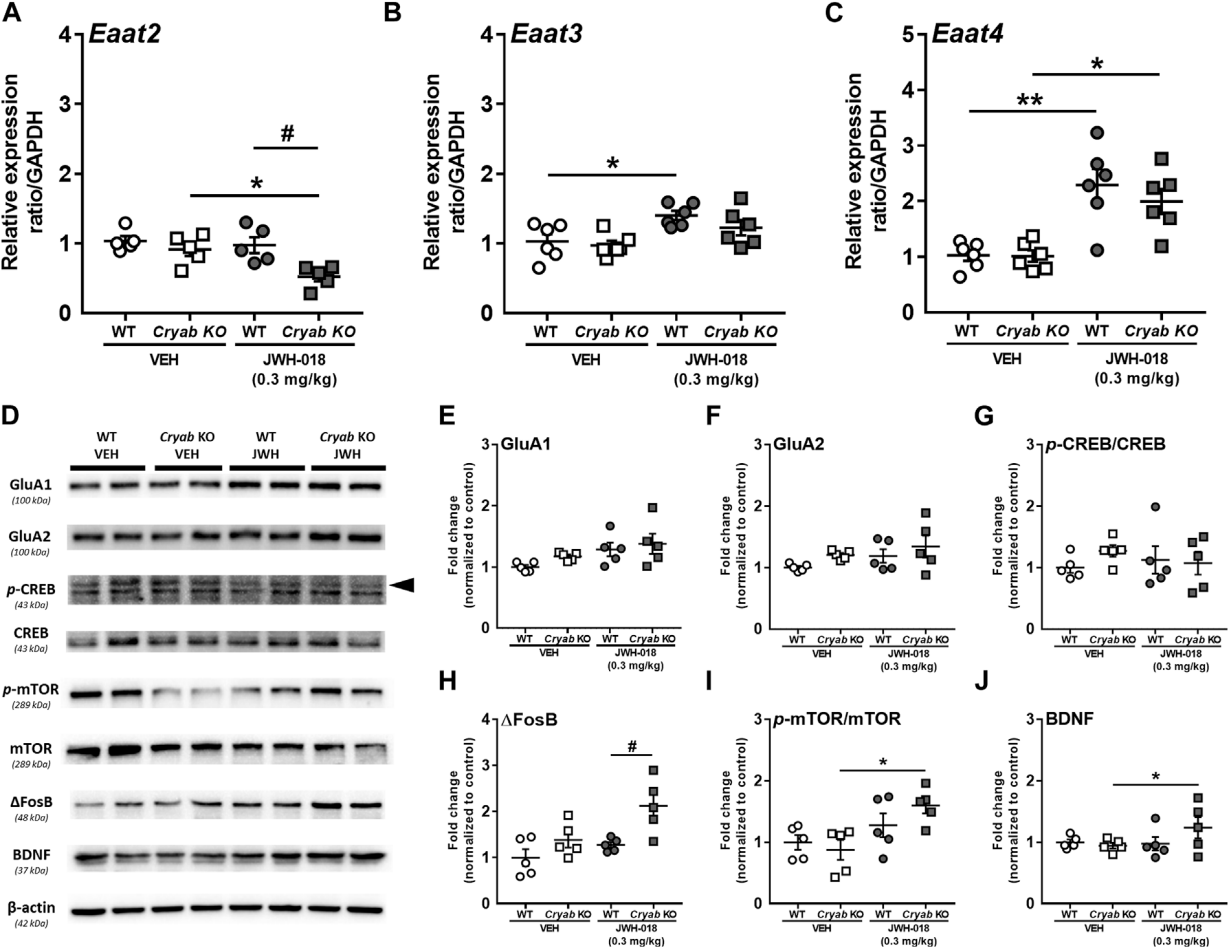
Glutamate transporter mRNA levels and plasticity-related protein expressions in wild-type (WT) and *Cryab* knockout (KO) mice following JWH-018 exposure. mRNA expression levels of **(A)** *Eaat2* **(B)** *Eaat3*, and **(C)** *Eaat4* in JWH-018-exposed mice was determined by quantitative real-time polymerase chain reaction. *n* = 5–6. **(D)** Representative blots showing the expression of target proteins in mice STR. Western blotting revealed the expression levels of **(E)** GluA1, **(F)** GluA2, **(G)** *p*-CREB/CREB, **(H)** ΔFosB, **(I)** *p*-mTOR/mTOR, and **(J)** BDNF in the STR of JWH-018-exposed mice. *n* = 4–5. Data expressed as mean ± S.E.M. *p < 0.05 and **p < 0.01 (vs. VEH; Tukey’s *post hoc* analysis). ^#^ *p* < 0.05 (vs. WT; Tukey’s *post hoc* analysis).

Glutamate reuptake in synapses is said to decrease, *via* GLT-1 downregulation, following an increase in cytokine expression and astrocyte activation (Boycott et al., 2008; Ramos et al., 2010), possibly altering glutamate concentration. Thus, we determined the mRNA expressions of glutamate transporter subtypes in JWH-018 exposed mice. Expression level of *Eaat2* mRNA (Figure 6A) was significantly decreased in *Cryab KO* mice and was lower than WT mice (genotype: F_1,16_ = 10.4; *p* < 0.01; treatment: F_1,16_ = 6.51; *p* < 0.05) after repeated treatment of JWH-018. *Eaat3* (Figure 6B) and *Eaat4* (Figure 6C) mRNA levels were both increased in WT mice (*Eaat3*: F_1,20_ = 12.5; *p* < 0.01, *Eaat4*: F_1,20_ = 32.5; *p* < 0.001), while only *Eaat4* mRNA was upregulated in *Cryab* KO mice after repeated JWH-018 administration.

Alterations in glutamate neurotransmission have been previously associated with the development of drug addiction, including those induced by psychostimulants (Kalivas, 2007) and cannabinoids (Cohen et al., 2019). These are generally characterized by changes in glutamate receptors and markers of synaptic plasticity in brain areas mediating addiction-related behaviors (Kauer and Malenka, 2007; Park et al., 2015). In here, we determined the expressions of glutamate receptors and synaptic plasticity markers in the STR (Figure 6D). GluA1 (Figure 6E; F_1,16_ = 5.92; *p* < 0.05), but not GluA2 (Figure 6F), expression showed significant differences among treatment groups, but only an increased trend in JWH-018-exposed *Cryab* KO mice can be observed. While *p*-CREB expressions of mice (Figure 6G) exhibited no significant variations among groups, ΔFosB expression (Figure 6H; genotype: F_1,16_ = 7.69; *p* < 0.01; treatment: F_1,16_ = 11.4; *p* < 0.05) was significantly higher in JWH-018-exposed *Cryab* KO mice than WT. *p*-mTOR (Figure 6I; treatment: F_1,16_ = 10.6; *p* < 0.01) and BDNF (Figure 6J genotype/treatment interaction: F_1,15_ = 5.21; *p* < 0.05) levels were increased in *Cryab* KO mice after repeated JWH-018 administration.

### 3.6 *Cryab* KO mice displayed higher NF-κB immunoreactivity in the STR following JWH-018 SA

**FIGURE 7:**
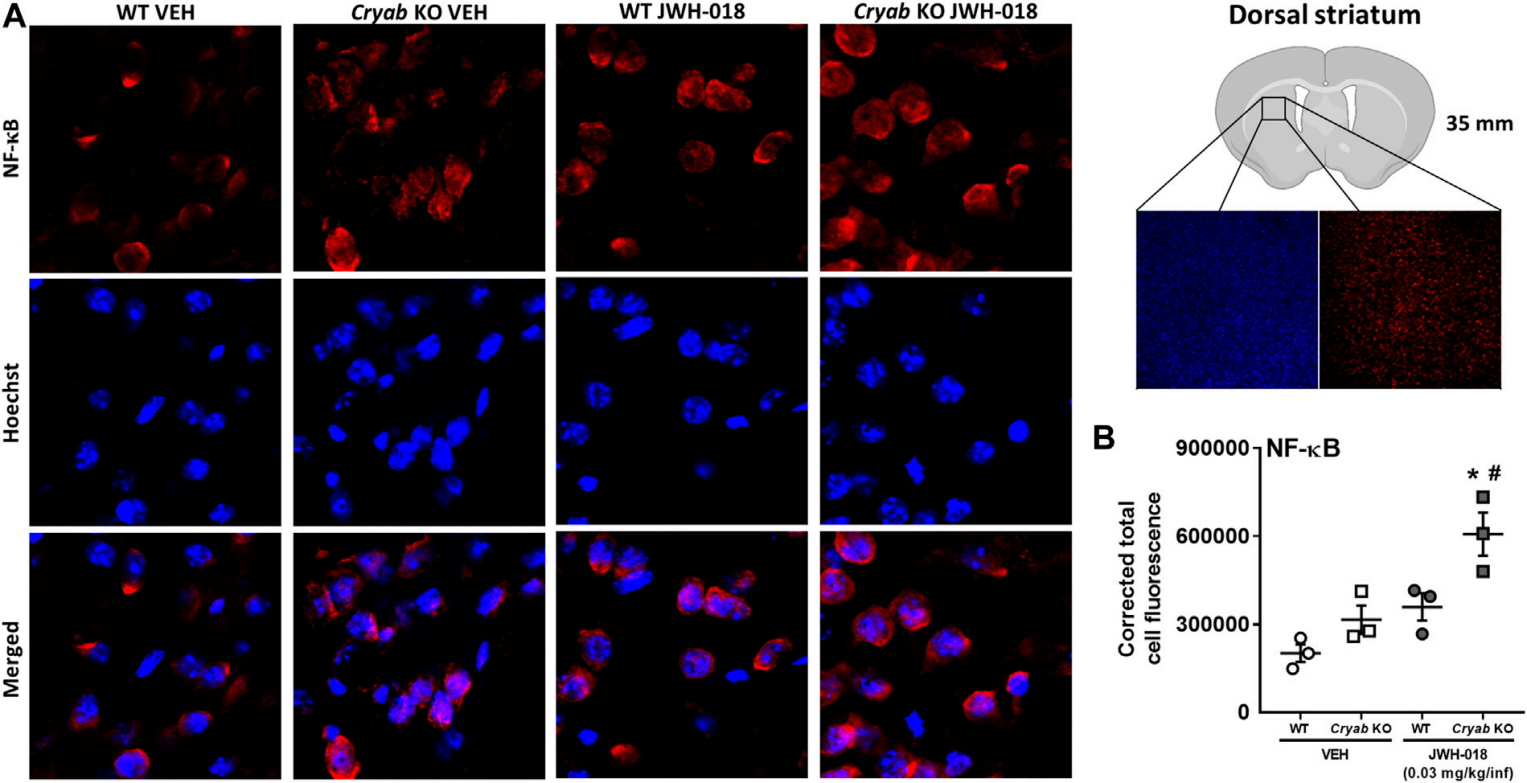
NF-κB immunoreactivity in mice from JWH-018 self-administration. **(A)** Immunofluorescence analysis of NF-κB and Hoechst staining in VEH- or JWH-018-exposed WT and *Cryab* KO mice at ×150 magnification (merged images are shown). **(B)** Analysis through ImageJ determined the corrected total cell fluorescence of NF-κB in mice following JWH-018 self-administration. *n* = 3. Data expressed as mean ± S.E.M. *p < 0.05 (vs. VEH; Tukey’s *post hoc* analysis). ^#^ *p* < 0.05 (vs. WT; Tukey’s *post hoc* analysis).

To verify the potential involvement of increased NF-κB expression in the manifestation of cannabinoid-induced addiction-related behaviors, we determined the striatal NF-κB expression of mice that underwent JWH-018 SA. NF-κB CTCF (Figure 7B) was significantly greater in *Cryab* KO mice (genotype: F_1,8_ = 12.2; *p* < 0.01; treatment: F_1,8_ = 18.8; *p* < 0.01) compared to their WT and VEH counterparts after JWH-018 SA.

## 4 Discussion

*Cryab* KO mice displayed generally similar baseline behavior with WT mice (Figures 2C, E–J), other than the marked hypoactivity in OFT (Figures 2A, B). This kind of phenotype was previously observed to occur alongside other aberrant behaviors, such as anxiety (Wood et al., 1987), depression (Ota et al., 2018), or autism spectrum disorder (Wang et al., 2018; Angelakos et al., 2019). Animal models for depression, which were also observed to possess inherently low spontaneous locomotor activity (Skalisz et al., 2002; Rygula et al., 2005; Ota et al., 2018), demonstrated increased sensitivity to cocaine (Lepsch et al., 2005; Ribeiro Do Couto et al., 2009; Xu et al., 2020) and nicotine (Cruz et al., 2008). The hypoactive behavior of *Cryab* KO mice might then indicate a possibility for them to likewise exhibit aberrant responses to psychoactive drugs. Furthermore, *Cryab* KO and WT mice might differentially respond to addictive drugs also due to their divergent baseline EEG recordings (Figure 2K). Human EEG data demonstrated persistent changes in the delta, alpha, and gamma waves of subjects repeatedly exposed to addictive drugs, such as cocaine (Reid et al., 2006) and marijuana (Herning et al., 2008), indicating the involvement of EEG waves in the neuropsychological effects of addictive drugs. Together, the hypoactivity and baseline EEG of *Cryab* KO mice might render them potentially sensitive to psychoactive substances, probably including synthetic cannabinoids. We then subsequently investigated the behavioral responses of *Cryab* KO mice to the effects of the synthetic cannabinoid JWH-018 using paradigms that generally indicate the rewarding and reinforcing effects of drugs.

*Cryab* KO mice exhibited potentially higher sensitivity to the addiction-related effects of JWH-018, as evidenced by their increased responses in intravenous SA (Figures 3A–C) and CPP (Figure 3E) tests compared to WT mice. While several studies have reported the addiction-like effects of synthetic cannabinoids, including JWH-018 (Hur et al., 2021), in healthy rodent subjects (Cha et al., 2014; de Luca et al., 2015), some cannabinoids still do not exhibit significant abuse potential in animal behavioral paradigms (Tampus et al., 2015; Bilel et al., 2019). This suggests the involvement of complex neurological mechanisms (that mediate the addictive effects of cannabinoids) other than the typical mesolimbic dopaminergic pathway. The results of the SA and CPP tests were consistent with this hypothesis, as WT mice did not exhibit significant preference or tendency for self-administering JWH-018 at 0.03 mg/kg/infusion or 0.3 mg/kg, unlike with previous studies. This discrepancy among studies investigating intravenous JWH-018 SA in WT mice might be attributed primarily to methodological differences, given that some studies required long-term exposure of mice in JWH-018 SA (de Luca et al., 2015), with some using adolescent mice with potentially different drug sensitivity (Margiani et al., 2022). However, the fact that *Cryab* KO mice exhibited modest SA responses and place preference for JWH-018 indicated cannabinoid-induced addiction-like behaviors, suggesting cannabinoid abuse susceptibility.

EEG recordings revealed significantly different gamma-wave power changes in WT and *Cryab* KO mice following repeated exposures to JWH-018 (Figures 3F–H). Gamma oscillations are involved in both cognition and perceptive functions and may be mediated by CB_1_ receptors *via* GABA interneurons (Skosnik et al., 2012), indicating the potential involvement of gamma-wave alterations in cannabinoid-mediated effects, which may include addiction. Previous studies have also reported that repeated exposure to various addictive drugs increases gamma-wave production (Abiero et al., 2020; Custodio et al., 2020), which was also observed in *Cryab* KO mice after repeated JWH-018 exposure. However, gamma-wave production is generally associated with attention and perception (Müller et al., 2000; Tallon-Baudry et al., 2005), which may implicate the JWH-018-induced increase in *Cryab* KO mice gamma-wave power as an improvement in receptivity and cognition. While this may not entirely indicate an addiction response or vulnerability, the divergent changes in gamma-wave power between WT and *Cryab* KO mice might still indicate different neurophysiological modifications in brain substrates that may contribute to the observed disparity in the cannabinoid-induced, addiction-like responses between genotypes. Thus far, behavioral and EEG data suggest the *Cryab* KO mice to be a potential animal model of cannabinoid abuse susceptibility.

As previously mentioned, psychoactive cannabinoids generally activate CB_1_ receptors on GABA/glutamatergic neurons located in the VTA and NAC, thus modulating the release of GABA/glutamate (Cristino et al., 2020; Spanagel, 2020) and influencing dopamine-mediated activities, such as addiction responses. Thus, a dysregulation in these pathways may influence cannabinoid-induced responses. Endocannabinoid-related mRNA expression levels (Figures 4A–H) suggest a possible downregulation of endocannabinoid signaling in WT and *Cryab* KO mice VTA, and perhaps also in WT mice NAC, after repeated JWH-018 treatment. While frequent exposure to cannabinoids potentially downregulates cannabinoid receptors in some brain regions (Scherma et al., 2016; van de Giessen et al., 2017; Kesner and Lovinger, 2021), similar to our qRT-PCR data, comparable JWH-018-induced mRNA alterations were detected between *Cryab* KO and WT mice, indicating possibly similar effects on dopaminergic signaling. Since alterations in cannabinoid receptor-encoding gene expressions may indicate GABA/glutamate signaling adaptations and dopaminergic activity dysregulation (Maldonado et al., 2011; Colizzi et al., 2016; Zehra et al., 2018; Pintori et al., 2021), comparable modifications in both *Cryab* KO and WT mice might suggest similarities in the expression of cannabinoid-induced behaviors. As this was not the case, given the SA and CPP results, the disparity between the JWH-018-induced behavioral responses of WT and *Cryab* KO mice might involve divergent modifications in dopaminergic signaling.

Drug addiction is frequently attributed to alterations in dopamine neurotransmission, along with upregulated accumbal dopamine levels (Fleckenstein et al., 2007; MacNicol, 2017; Jiménez-González et al., 2022). Expressions of dopamine-related mRNA in the NAC of JWH-018-exposed mice (Figures 4I–L) suggest a possible dampening of dopaminergic activity. The same phenomenon was also observed in previous studies showing the blunting of dopaminergic activity following chronic cannabinoid exposure (van de Giessen et al., 2017; Kesner and Lovinger, 2021). It was previously suggested that genes influencing dopamine availability tend to also downregulate following chronic cannabinoid treatments (Perdikaris et al., 2018). The *Dat* and *Vmat2* downregulation in WT mice may suggest a compensatory response to promote dopamine availability, which is a phenomenon also found during drug tolerance and in chronic methamphetamine users (Wilson et al., 1996; Graves et al., 2021). Although there are only limited studies describing the effect of cannabinoids on *Vmat2* mRNA, its downregulation may denote possible dopamine release dysregulation (German et al., 2015) in WT mice in response to cannabinoids. One study suggested that reduced *Vmat2* mRNA expression may lead to inhibition of vesicular dopamine and potentially increase cytosolic dopamine levels (Sun et al., 2014). The lack of such changes in *Cryab* KO mice may imply their resilience to cannabinoid-induced dopaminergic alterations, further suggesting dopamine-independent mechanisms mediating their higher cannabinoid sensitivity. Nevertheless, downregulation of endocannabinoid signaling may have contributed to possible dopaminergic desensitization following repeated JWH-018 administration, which is a potential theory behind cannabinoid addiction pathophysiology (Zehra et al., 2018). On the other hand, the involvement of dopamine mediation might still not be ruled out completely. Studies demonstrating dopamine level upregulation after acute cannabinoid administration (de Luca et al., 2015; Ossato et al., 2017; Pintori et al., 2021), along with our ELISA results following acute JWH-018 treatment (Figure 4M), may still implicate transient dopamine increments in addiction-related brain regions to the addictive properties of cannabinoids. However, probably due to the absence of sustained dopaminergic activation *via* endocannabinoid signaling downregulation, some cannabinoids may have appeared to lack robust abuse potential following prolonged exposure (Cha et al., 2014; Tampus et al., 2015), as opposed to recognized abused drugs that show persistent increases in dopamine concentration and activity even after repeated exposures (Nestby et al., 1997; Cadoni et al., 2000), resulting in evident abuse liability. Therefore, the differential responses between *Cryab* KO and WT mice to the addiction-inducing effects of JWH-018 may implicate the involvement of other neurological mechanisms beyond the endocannabinoid or dopaminergic systems.

Studies have suggested the involvement of CRYAB and cannabinoids in the anti-inflammatory action of the PI3K-AKT-GSK3 pathway (Ozaita et al., 2007; Xu et al., 2013; Zhu et al., 2015), which may also modulate the expression of NF-κB (Bathina and Das, 2018), a transcription factor broadly associated with immune system-related and inflammation-related gene regulation (Beurel et al., 2015; Liu et al., 2017). Several factors (Beurel et al., 2015; Liu et al., 2017; Feng and Lu, 2021), including some heat shock proteins (Schell et al., 2005), were reported to potentially influence neuroinflammation by regulating NF-κB expression/activity, and when modulated, altered complex behaviors such as addiction (Rodrigues et al., 2014; Nennig and Schank, 2017; Morcuende et al., 2021). More recently, CRYAB was described to potentially regulate NF-κB nuclear translocation (Shao et al., 2013; Qiu et al., 2016), which is essential for inflammatory cytokine and chemokine transcription (Somade et al., 2019). Based on the possible correlation between cannabinoid addiction and neuroinflammation, CRYAB, *via* NF-κB, may potentially influence addiction-related behaviors. Increased CRYAB (Figure 5B) and negligible changes along the PI3K-AKT-GSK3 pathway (Figures 5C–F) in JWH-018-exposed WT mice may imply the occurrence of only minor neuroinflammation. In contrast, JWH-018-exposed *Cryab* KO mice seemed to suggest potential downstream neuroinflammatory alterations, given by increased GSK-3β (Figure 5F) and NF-κB (Figure 5G) expressions. From this observation, functional CRYAB expression might have a role in the anti-inflammatory effect of cannabinoids, as the presence or absence of CRYAB resulted in significant genotype differences in the expression of NF-κB following JWH-018 exposure. Furthermore, NF-κB expression and translocation may also influence neuroinflammation by mediating inflammatory cytokine gene transcription and astroglia activation (Young et al., 2011; Liu et al., 2017). While JWH-018 exposure induced no changes in WT mice, the higher expression of glial fibrillary acidic protein (GFAP; Figure 5H) and IL-6 (Figure 5K) in *Cryab* KO mice may indicate a higher state of neuroinflammation compared to WT mice, which may have been a consequence of increased NF-κB expression following repeated JWH-018 administration. Intriguingly, this had no effect on TNF-α and IL-1β expressions, unlike in previous reports (Nennig and Schank, 2017), which could suggest that the alteration of CRYAB expression may have specificity to IL-6 transcription. Our results contradict some previous studies, given that cannabinoids are understood to be anti-inflammatory and JWH-018 seemed to potentially induce inflammation in *Cryab* KO mice. However, according to previous studies (Pintori et al., 2021; Margiani et al., 2022), JWH-018 may potentially promote neuroinflammation in mice following previous repeated exposures. Thus, the absence of CRYAB might have exacerbated the neuroinflammatory effect of JWH-018 in *Cryab* KO mice, resulting in the observed alterations in NF-κB, GFAP, and IL-6 expressions, given also that CRYAB, possessing an anti-inflammatory function, might have rendered the *Cryab* KO mice susceptible to inflammatory insults.

Increased neuroinflammation, specifically due to increased cytokine and astrocyte activation (Boycott et al., 2008), may result to the downregulation of glutamate transporter (GLT-1) (Ramos et al., 2010), potentially increasing synaptic glutamate concentration. Glutamate reuptake was also reported to be reduced in the STR of cocaine-exposed rodents (Parikh et al., 2014; Smaga et al., 2020), potentially increasing the availability of glutamate in the synapse. Taking together the alterations in glutamate transporter mRNA (Figures 6A–C), there might be an overall decrease in glutamate reuptake in *Cryab* KO mice, suggesting potentially higher glutamate levels in the STR of *Cryab* KO mice, compared to that in WT after JWH-018 exposure, which may have implications in glutamate-mediated long-term potentiation. While glutamate-mediated cannabinoid addiction may entail their regulation of glutamate activation of medium spiny neurons in the NAC (Cohen et al., 2019), other drugs of abuse elicit glutamate-mediated addiction through long-term potentiation of the glutamatergic synapse (LaLumiere and Kalivas, 2008; Gipson et al., 2014; Griffin et al., 2015) *via* potential neuroinflammatory mechanisms (Hutchinson et al., 2012; Zhu et al., 2018). Similarly, the expressions of synaptic plasticity markers in *Cryab* KO mice (Figures 6G–J) may suggest the occurrence of neuronal adaptations following JWH-018 exposure, which may have contributed to the development and maintenance of drug-induced addiction (Tsai, 2007; Post and Kalivas, 2013; Zhang et al., 2014; Sutton and Caron, 2015; Ucha et al., 2020; García-García et al., 2021) in *Cryab* KO mice. While it is still uncertain whether these changes were indeed a result of increased glutamate receptor activation, given the lack of genotype variations in GluA1 and GluA2 expressions, although with marked differences among groups (Figures 6E, F), the lack of CRYAB during JWH-018 administration might still ultimately induce positive effects on drug cue reinforcement. Since glutamate receptor activation may reinforce addiction-related behaviors through postsynaptic alterations (Gass and Olive, 2008; Joffe et al., 2014; Nennig and Schank, 2017), the greater striatal synaptic adaptations in drug-exposed *Cryab* KO mice may have induced stronger improved habit-associated learning from neuroinflammation-mediated long-term potentiation. This can be supported by NF-κB fluorescent signaling in *Cryab* KO mice from JWH-018 SA (Figures 7A, B), implying that upregulated NF-κB expression might indeed be involved in the manifestation of cannabinoid-induced addiction-like behaviors in *Cryab* KO mice, even through various means of JWH-018 exposure (daily repeated treatment or self-administered). Therefore, functional CRYAB deficiency may have enhanced the occurrence of increased striatal synaptic plasticity *via* NF-κB-modulated neuroinflammation following JWH-018 exposure, potentially corroborating *Cryab* in neuroinflammation-mediated cannabinoid addiction.

**FIGURE 8:**
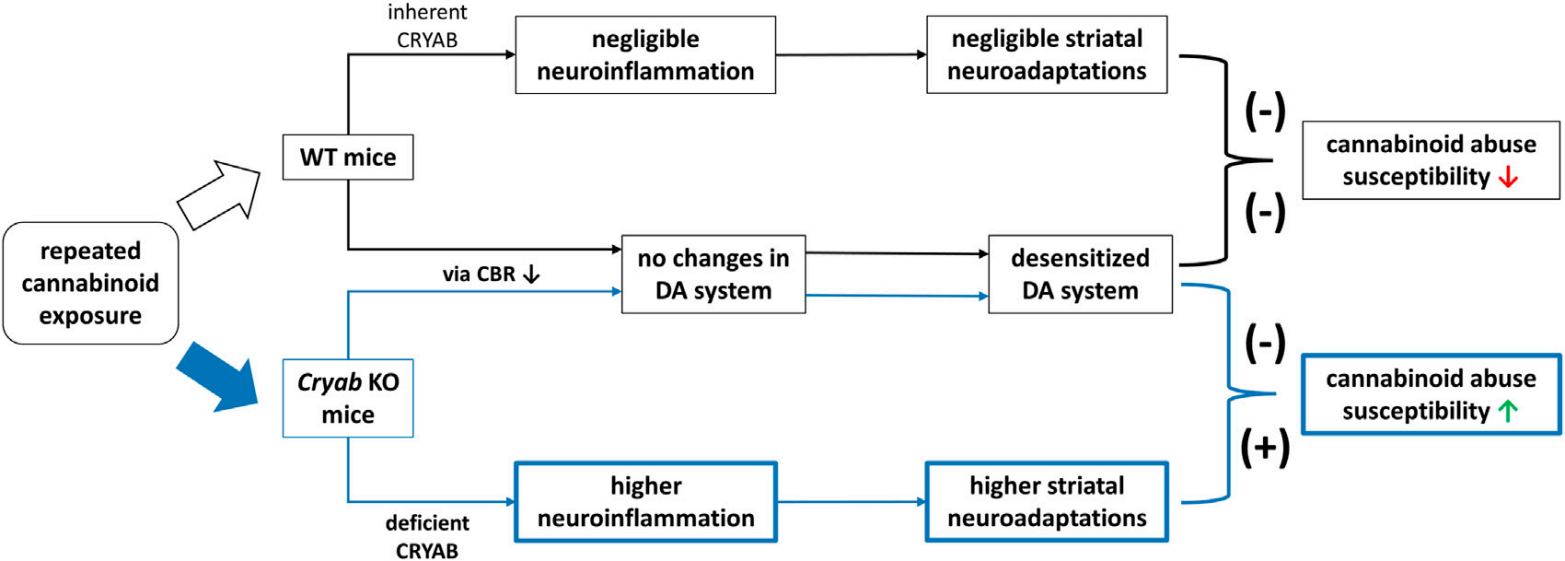
Schematic diagram for a proposed mechanism underlying the enhanced JWH-018 abuse susceptibility of *Cryab* knockout (KO) mice. Repeated administration of cannabinoids, such JWH-018, may result to a reduction in cannabinoid receptor (CBR) expression, leading to a desensitized dopamine (DA) system in both *Cryab* KO and wild-type (WT) mice. Due to CRYAB deficiency, repeated JWH-018 administration resulted in higher striatal neuroinflammation, leading to higher neuroadaptations. These changes may potentially contribute to the abuse susceptibility of *Cryab* KO mice to cannabinoids.

In conclusion, *Cryab* KO mice exhibit higher cannabinoid-induced addiction than WT mice. Their divergent responses to cannabinoids compared to WT may involve neuroinflammation-mediated synaptic plasticity, due to functional CRYAB deletion (Figure 8). Such behavior might not also involve genotype differences in cannabinoid-induced alterations in the endocannabinoid or dopaminergic systems. Given these findings, future studies are imperative to investigate the behavioral responses of *Cryab* KO mice to other types of cannabinoids to further verify the involvement of *Cryab* in general cannabinoid addiction. Taken together, the *Cryab* KO mice might represent one of the potential mechanisms for enhanced cannabinoid abuse susceptibility and be an efficient tool for screening the abuse potential of novel synthetic cannabinoids, contributing to more reliable and accurate evaluation regarding the potential dangers of these substances in humans.

## Data Availability

The original contributions presented in the study are included in the article/Supplementary Material, further inquiries can be directed to the corresponding authors.

## References

[B1] Abiero A. Botanas C. J. Custodio R. J. Sayson L. V. Kim M. Lee H. J. (2020). 4-MeO-PCP and 3-MeO-PCMo, new dissociative drugs, produce rewarding and reinforcing effects through activation of mesolimbic dopamine pathway and alteration of accumbal CREB, deltaFosB, and BDNF levels. Psychopharmacol. Berl. 237, 757–772. 10.1007/s00213-019-05412-y 31828394

[B2] Adinoff B. (2004). Neurobiologic processes in drug reward and addiction. Harv Rev. Psychiatry 12, 305–320. 10.1080/10673220490910844 15764467 PMC1920543

[B3] Angelakos C. C. Tudor J. C. Ferri S. L. Jongens T. A. Abel T. (2019). Home-cage hypoactivity in mouse genetic models of autism spectrum disorder. Neurobiol. Learn Mem. 165, 107000. 10.1016/J.NLM.2019.02.010 30797034 PMC6913530

[B4] Bathina S. Das U. N. (2018). Dysregulation of PI3K-Akt-mTOR pathway in brain of streptozotocin-induced type 2 diabetes mellitus in Wistar rats. Lipids Health Dis. 17, 168. 10.1186/s12944-018-0809-2 30041644 PMC6058366

[B5] Bayazit H. Selek S. Karababa I. F. Cicek E. Aksoy N. (2017). Evaluation of oxidant/antioxidant status and cytokine levels in patients with cannabis use disorder. Clin. Psychopharmacol. Neurosci. 15, 237–242. 10.9758/cpn.2017.15.3.237 28783932 PMC5565077

[B6] Beurel E. Grieco S. F. Jope R. S. (2015). Glycogen synthase kinase-3 (GSK3): Regulation, actions, and diseases. Pharmacol. Ther. 148, 114–131. 10.1016/j.pharmthera.2014.11.016 25435019 PMC4340754

[B7] Bilel S. Tirri M. Arfè R. Stopponi S. Soverchia L. Ciccocioppo R. (2019). Pharmacological and behavioral effects of the synthetic cannabinoid AKB48 in rats. Front. Neurosci. 13, 1163. 10.3389/fnins.2019.01163 31736697 PMC6831561

[B8] Botanas C. J. Perez Custodio R. J. Kim H. J. de la Pena J. B. Sayson L. V. Ortiz D. M. (2021). R (−)-methoxetamine exerts rapid and sustained antidepressant effects and fewer behavioral side effects relative to S (+)-methoxetamine. Neuropharmacology 193, 108619. 10.1016/j.neuropharm.2021.108619 34023336

[B9] Boycott H. E. Wilkinson J. A. Boyle J. P. Pearson H. A. Peers C. (2008). Differential involvement of TNFα in hypoxic suppression of astrocyte glutamate transporters. Glia 56, 998–1004. 10.1002/glia.20673 18381653

[B10] Cadoni C. Solinas M. di Chiara G. (2000). Psychostimulant sensitization: Differential changes in accumbal shell and core dopamine. Eur. J. Pharmacol. 388, 69–76. 10.1016/S0014-2999(99)00824-9 10657548

[B11] Calderwood S. K. Repasky E. A. Neckers L. Hightower L. E. (2019). The IXth CSSI international symposium on heat shock proteins in biology and medicine: Stress responses in health and disease: Alexandria old town, alexandria, Virginia, november 10–13, 2018. Cell. Stress Chaperones 24, 1–6. 10.1007/S12192-018-00966-W 30645757 PMC6363612

[B12] Cha H. J. Lee K. W. Song M. J. Hyeon Y. J. Hwang J. Y. Jang C. G. (2014). Dependence potential of the synthetic cannabinoids JWH-073, JWH-081, and JWH-210: *In vivo* and *in vitro* approaches. Biomol. Ther. Seoul. 22, 363–369. 10.4062/biomolther.2014.039 25143817 PMC4131522

[B13] Cohen K. Weizman A. Weinstein A. (2019). Modulatory effects of cannabinoids on brain neurotransmission. Eur. J. Neurosci. 50, 2322–2345. 10.1111/EJN.14407 30882962

[B14] Colizzi M. McGuire P. Pertwee R. G. Bhattacharyya S. (2016). Effect of cannabis on glutamate signalling in the brain: A systematic review of human and animal evidence. Neurosci. Biobehav Rev. 64, 359–381. 10.1016/j.neubiorev.2016.03.010 26987641

[B15] Cristino L. Bisogno T. di Marzo V. (2020). Cannabinoids and the expanded endocannabinoid system in neurological disorders. Nat. Rev. Neurol. 16, 9–29. 10.1038/s41582-019-0284-z 31831863

[B16] Cruz F. C. DeLucia R. Planeta C. S. (2008). Effects of chronic stress on nicotine-induced locomotor activity and corticosterone release in adult and adolescent rats. Addict. Biol. 13, 63–69. 10.1111/j.1369-1600.2007.00080.x 17850415

[B17] Custodio R. J. P. Botanas C. J. de la Peña J. B. dela Peña I. J. Kim M. Sayson L. V. (2018). Overexpression of the thyroid hormone-responsive (THRSP) gene in the striatum leads to the development of inattentive-like phenotype in mice. Neuroscience 390, 141–150. 10.1016/j.neuroscience.2018.08.008 30138648

[B18] Custodio R. J. P. Kim M. Sayson L. V. Lee H. J. Ortiz D. M. Kim B. N. (2021). Low striatal T3 is implicated in inattention and memory impairment in an ADHD mouse model overexpressing thyroid hormone-responsive protein. Commun. Biol. 4, 1101–1114. 10.1038/s42003-021-02633-w 34545202 PMC8452653

[B19] Custodio R. J. P. Kim M. Sayson L. V. Ortiz D. M. Buctot D. Lee H. J. (2022). Regulation of clock and clock-controlled genes during morphine reward and reinforcement: Involvement of the period 2 circadian clock. J. Psychopharmacol. 36, 875–891. 10.1177/02698811221089040 35486444

[B20] Custodio R. J. P. Sayson L. V. Botanas C. J. Abiero A. You K. Y. Kim M. (2020). 25B-NBOMe, a novel N-2-methoxybenzyl-phenethylamine (NBOMe) derivative, may induce rewarding and reinforcing effects via a dopaminergic mechanism: Evidence of abuse potential. Addict. Biol. 25, e12850. 10.1111/adb.12850 31749223

[B21] Dai A. Guo X. Yang X. Li M. Fu Y. Sun Q. (2022). Effects of the CRYAB gene on stem cell-like properties of colorectal cancer and its mechanism. J. Cancer Res. Ther. 18, 1328–1337. 10.4103/JCRT.JCRT_212_22 36204880

[B22] de Luca M. A. Bimpisidis Z. Melis M. Marti M. Caboni P. Valentini V. (2015). Stimulation of *in vivo* dopamine transmission and intravenous self-administration in rats and mice by JWH-018, a Spice cannabinoid. Neuropharmacology 99, 705–714. 10.1016/j.neuropharm.2015.08.041 26327678

[B23] de Luca M. A. Fattore L. (2018). Therapeutic use of synthetic cannabinoids: Still an open issue? Clin. Ther. 40, 1457–1466. 10.1016/j.clinthera.2018.08.002 30180974

[B24] dela Peña I. J. I. Botanas C. J. de la Peña J. B. Custodio R. J. dela Peña I. Ryoo Z. Y. (2019). The atxn7-overexpressing mice showed hyperactivity and impulsivity which were ameliorated by atomoxetine treatment: A possible animal model of the hyperactive-impulsive phenotype of adhd. Prog. Neuropsychopharmacol. Biol. Psychiatry 88, 311–319. 10.1016/j.pnpbp.2018.08.012 30125623

[B25] Delis F. Polissidis A. Poulia N. Justinova Z. Nomikos G. G. Goldberg S. R. (2017). Attenuation of cocaine-induced conditioned place preference and motor activity via cannabinoid CB2 receptor agonism and cb1 receptor antagonism in rats. Int. J. Neuropsychopharmacol. 20, 269–278. 10.1093/ijnp/pyw102 27994006 PMC5408977

[B26] Diao X. Huestis M. A. (2019). New synthetic cannabinoids metabolism and strategies to best identify optimal marker metabolites. Front. Chem. 7, 109. 10.3389/fchem.2019.00109 30886845 PMC6409358

[B27] Drug Enforcement Administration (2015). Drugs of abuse 2015 edition: A dea resource guide. Springfield: Drug Enforcement Administration.

[B28] Fagundo A. B. de la Torre R. Jiménez-Murcia S. Agüera Z. Pastor A. Casanueva F. F. (2013). Modulation of the endocannabinoids N-arachidonoylethanolamine (AEA) and 2-arachidonoylglycerol (2-AG) on executive functions in humans. PLoS One 8, e66387. 10.1371/journal.pone.0066387 23840456 PMC3686875

[B29] Feng Y. Lu Y. (2021). Immunomodulatory effects of dopamine in inflammatory diseases. Front. Immunol. 12, 663102. 10.3389/fimmu.2021.663102 33897712 PMC8063048

[B30] Fleckenstein A. E. Volz T. J. Riddle E. L. Gibb J. W. Hanson G. R. (2007). New insights into the mechanism of action of amphetamines. Annu. Rev. Pharmacol. Toxicol. 47, 681–698. 10.1146/annurev.pharmtox.47.120505.105140 17209801

[B31] García-García F. Priego-Fernández S. López-Muciño L. A. Acosta-Hernández M. E. Peña-Escudero C. (2021). Increased alcohol consumption in sleep-restricted rats is mediated by delta FosB induction. Alcohol 93, 63–70. 10.1016/J.ALCOHOL.2021.02.004 33662520

[B32] Gass J. T. Olive M. F. (2008). Glutamatergic substrates of drug addiction and alcoholism. Biochem. Pharmacol. 75, 218–265. 10.1016/J.BCP.2007.06.039 17706608 PMC2239014

[B33] German C. L. Baladi M. G. McFadden L. M. Hanson G. R. Fleckenstein A. E. (2015). Regulation of the dopamine and vesicular monoamine transporters: Pharmacological targets and implications for disease. Pharmacol. Rev. 67, 1005–1024. 10.1124/pr.114.010397 26408528 PMC4630566

[B34] Gipson C. D. Kupchik Y. M. Kalivas P. W. (2014). Rapid, transient synaptic plasticity in addiction. Neuropharmacology 76, 276–286. 10.1016/j.neuropharm.2013.04.032 23639436 PMC3762905

[B35] Graves S. M. Schwarzschild S. E. Tai R. A. Chen Y. Surmeier D. J. (2021). Mitochondrial oxidant stress mediates methamphetamine neurotoxicity in substantia nigra dopaminergic neurons. Neurobiol. Dis. 156, 105409. 10.1016/j.nbd.2021.105409 34082123 PMC8686177

[B36] Griffin W. C. Ramachandra V. S. Knackstedt L. A. Becker H. C. (2015). Repeated cycles of chronic intermittent ethanol exposure increases basal glutamate in the nucleus accumbens of mice without affecting glutamate transport. Front. Pharmacol. 6, 27. 10.3389/fphar.2015.00027 25755641 PMC4337330

[B37] Guo Y. S. Liang P. Lu S. Z. Chen R. Yin Y. Zhou J. (2019). Extracellular αB-crystallin modulates the inflammatory responses. Biochem. Biophys. Res. Commun. 508, 282–288. 10.1016/j.bbrc.2018.11.024 30497777

[B38] Hammond L. (2014). Measuring cell fluorescence using ImageJ — the open Lab book v1.0. The open Lab book. Available at: https://theolb.readthedocs.io/en/latest/imaging/measuring-cell-fluorescence-using-imagej.html (Accessed October 4, 2022).

[B39] Herning R. I. Better W. Cadet J. L. (2008). EEG of chronic marijuana users during abstinence: Relationship to years of marijuana use, cerebral blood flow and thyroid function. Clin. Neurophysiol. 119, 321–331. 10.1016/j.clinph.2007.09.140 18065267 PMC2234454

[B40] Herrero M. J. Domingo-Salvany A. Torrens M. Brugal M. T. de Hoz L. D. L. F. Gómez R. B. (2008). Psychiatric comorbidity in young cocaine users: Induced versus independent disorders. Addiction 103, 284–293. 10.1111/j.1360-0443.2007.02076.x 18199307

[B41] Huffman J. W. Zengin G. Wu M. J. Lu J. Hynd G. Bushell K. (2005). Structure-activity relationships for 1-alkyl-3-(1-naphthoyl)indoles at the cannabinoid CB 1 and CB 2 receptors: Steric and electronic effects of naphthoyl substituents. New highly selective CB 2 receptor agonists. Bioorg Med. Chem. 13, 89–112. 10.1016/j.bmc.2004.09.050 15582455

[B42] Hur K. H. Ma S. X. Lee B. R. Ko Y. H. Seo J. Y. Ryu H. W. (2021). Abuse potential of synthetic cannabinoids: Am-1248, cb-13, and pb-22. Biomol. Ther. Seoul. 29, 384–391. 10.4062/biomolther.2020.212 33935046 PMC8255142

[B43] Hutchinson M. R. Northcutt A. L. Hiranita T. Wang X. Lewis S. S. Thomas J. (2012). Opioid activation of toll-like receptor 4 contributes to drug reinforcement. Soc. Neurosci. 32, 11187–11200. 10.1523/JNEUROSCI.0684-12.2012 PMC3454463 22895704

[B44] Hyatt W. S. Fantegrossi W. E. (2014). Δ9-THC exposure attenuates aversive effects and reveals appetitive effects of K2/’Spice’ constituent JWH-018 in mice. Behav. Pharmacol. 25, 253–257. 10.1097/FBP.0000000000000034 24625557 PMC4157458

[B45] Javed H. Azimullah S. Haque M. E. Ojha S. K. (2016). Cannabinoid type 2 (CB2) receptors activation protects against oxidative stress and neuroinflammation associated dopaminergic neurodegeneration in rotenone model of Parkinson’s disease. Front. Neurosci. 10, 321. 10.3389/fnins.2016.00321 27531971 PMC4969295

[B46] Jiménez-González A. Gómez-Acevedo C. Ochoa-Aguilar A. Chavarría A. (2022). The role of glia in addiction: Dopamine as a modulator of glial responses in addiction. Cell. Mol. Neurobiol. 42, 2109–2120. 10.1007/s10571-021-01105-3 34057683 PMC11421599

[B47] Joffe M. Grueter C. A. Grueter B. A. (2014). Biological substrates of addiction. Wiley Interdiscip. Rev. Cogn. Sci. 5, 151–171. 10.1002/wcs.1273 24999377 PMC4078878

[B48] Kabir Z. D. Lee A. S. Rajadhyaksha A. M. (2016). L-Type Ca2+ channels in mood, cognition and addiction: Integrating human and rodent studies with a focus on behavioural endophenotypes. J. Physiology 594, 5823–5837. 10.1113/JP270673 PMC5063939 26913808

[B49] Kalivas P. W. (2007). Cocaine and amphetamine-like psychostimulants: Neuro circuitry and glutamate neuroplasticity. Dialogues Clin. Neurosci. 9, 389–397. 10.31887/DCNS.2007.9.4/PKALIVAS 18286799 PMC3202508

[B50] Kauer J. A. Malenka R. C. (2007). Synaptic plasticity and addiction. Nat. Rev. Neurosci. 8;(11), 844–858. 10.1038/nrn2234 17948030

[B51] Kesner A. J. Lovinger D. M. (2021). Cannabis use, abuse, and withdrawal: Cannabinergic mechanisms, clinical, and preclinical findings. J. Neurochem. 157, 1674–1696. 10.1111/jnc.15369 33891706 PMC9291571

[B52] Kim M. Custodio R. J. Botanas C. J. de la Peña J. B. Sayson L. V. Abiero A. (2019). The circadian gene, Per2, influences methamphetamine sensitization and reward through the dopaminergic system in the striatum of mice. Addict. Biol. 24, 946–957. 10.1111/adb.12663 30091820

[B53] Kinsey S. G. Mahadevan A. Zhao B. Sun H. Naidu P. S. Razdan R. K. (2011). The CB2 cannabinoid receptor-selective agonist O-3223 reduces pain and inflammation without apparent cannabinoid behavioral effects. Neuropharmacology 60, 244–251. 10.1016/j.neuropharm.2010.09.004 20849866 PMC3021987

[B54] Kohno M. Link J. Dennis L. E. McCready H. Huckans M. Hoffman W. F. (2019). Neuroinflammation in addiction: A review of neuroimaging studies and potential immunotherapies. Pharmacol. Biochem. Behav. 179, 34–42. 10.1016/j.pbb.2019.01.007 30695700 PMC6637953

[B55] Kozela E. Pietr M. Juknat A. Rimmerman N. Levy R. Vogel Z. (2010). Cannabinoids Delta(9)-tetrahydrocannabinol and cannabidiol differentially inhibit the lipopolysaccharide-activated NF-kappaB and interferon-beta/STAT proinflammatory pathways in BV-2 microglial cells. J. Biol. Chem. 285, 1616–1626. 10.1074/jbc.M109.069294 19910459 PMC2804319

[B56] Krasnova I. N. Justinova Z. Cadet J. L. (2016). Methamphetamine addiction: Involvement of CREB and neuroinflammatory signaling pathways. Psychopharmacol. Berl. 233, 1945–1962. 10.1007/s00213-016-4235-8 PMC5627363 26873080

[B57] Kuipers H. F. Yoon J. van Horssen J. Han M. H. Bollyky P. L. Palmer T. D. (2017). Phosphorylation of αB-crystallin supports reactive astrogliosis in demyelination. Proc. Natl. Acad. Sci. U. S. A. 114, E1745–E1754. 10.1073/PNAS.1621314114/SUPPL_FILE/PNAS.1621314114.SD01.XLSX 28196893 PMC5338510

[B58] LaLumiere R. T. Kalivas P. W. (2008). Glutamate release in the nucleus accumbens core is necessary for heroin seeking. J. Neurosci. 28, 3170–3177. 10.1523/JNEUROSCI.5129-07.2008 18354020 PMC6670700

[B59] Le Boisselier R. Alexandre J. Lelong-Boulouard V. Debruyne D. (2017). Focus on cannabinoids and synthetic cannabinoids. Clin. Pharmacol. Ther. 101, 220–229. 10.1002/cpt.563 27861784

[B60] Lepsch L. B. Gonzalo L. A. Magro F. J. B. Delucia R. Scavone C. Planeta C. S. (2005). Exposure to chronic stress increases the locomotor response to cocaine and the basal levels of corticosterone in adolescent rats. Addict. Biol. 10, 251–256. 10.1080/13556210500269366 16109586

[B61] Leung J. Chan G. C. K. Hides L. Hall W. D. (2020). What is the prevalence and risk of cannabis use disorders among people who use cannabis? A systematic review and meta-analysis. Addict. Behav. 109, 106479. 10.1016/j.addbeh.2020.106479 32485547

[B62] Li R. Fukumori R. Takeda T. Song Y. Morimoto S. Kikura-Hanajiri R. (2019). Elevation of endocannabinoids in the brain by synthetic cannabinoid JWH-018: Mechanism and effect on learning and memory. Sci. Rep. 9, 9621. 10.1038/S41598-019-45969-4 31270353 PMC6610139

[B63] Lin X. Dhopeshwarkar A. S. Huibregtse M. MacKie K. Hohmann A. G. (2018). Slowly signaling G protein-biased CB2 cannabinoid receptor agonist LY2828360 suppresses neuropathic pain with sustained efficacy and attenuates morphine tolerance and dependence. Mol. Pharmacol. 93, 49–62. 10.1124/mol.117.109355 29192123 PMC5749492

[B64] Liu T. Zhang L. Joo D. Sun S. C. (2017). NF-κB signaling in inflammation. Signal Transduct. Target Ther. 2, 17023. 10.1038/sigtrans.2017.23 29158945 PMC5661633

[B65] Liu Y. Chen Y. Fraga-González G. Szpak V. Laverman J. Wiers R. W. (2022). Resting-state EEG, substance use and abstinence after chronic use: A systematic review. Clin. EEG Neurosci. 53, 344–366. 10.1177/15500594221076347 35142589

[B66] MacDonald K. Pappas K. (2016). WHY not pot?: A review of the brain-based risks of cannabis. Innov. Clin. Neurosci. 13, 13–22. Available at: https://pubmed.ncbi.nlm.nih.gov/27354924/ (Accessed October 17, 2022). PMC4911936 27354924

[B67] MacNicol B. (2017). The biology of addiction. Can. J. Anesth. 64, 141–148. 10.1007/s12630-016-0771-2 27837404

[B68] Maldonado R. Berrendero F. Ozaita A. Robledo P. (2011). Neurochemical basis of cannabis addiction. Neuroscience 181, 1–17. 10.1016/j.neuroscience.2011.02.035 21334423

[B69] Malyshevskaya O. Aritake K. Kaushik M. K. Uchiyama N. Cherasse Y. Kikura-Hanajiri R. (2017). Natural (Δ9-THC) and synthetic (JWH-018) cannabinoids induce seizures by acting through the cannabinoid CB1 receptor. Sci. Rep. 7, 10516. 10.1038/s41598-017-10447-2 28874764 PMC5585372

[B70] Margiani G. Castelli M. P. Pintori N. Frau R. Ennas M. G. Orrù V. (2022). Adolescent self-administration of the synthetic cannabinoid receptor agonist JWH-018 induces neurobiological and behavioral alterations in adult male mice. Psychopharmacology 239:(10), 3083–3102. 10.1007/S00213-022-06191-9 35943523 PMC9481487

[B71] Ministry of Food and Drug Safety (2021). Development of sensitized screening method for addiction/dependence of abuse drugs. MFDS. Available at: https://scienceon.kisti.re.kr/commons/util/originalView.do?cn=TRKO202200005149&dbt=TRKO&rn= (Accessed February 20, 2023).

[B72] Morcuende A. Navarrete F. Nieto E. Manzanares J. Femenía T. (2021). Inflammatory biomarkers in addictive disorders. Biomolecules 11, 1824. 10.3390/biom11121824 34944470 PMC8699452

[B73] Müller M. M. Gruber T. Keil A. (2000). Modulation of induced gamma band activity in the human EEG by attention and visual information processing. Int. J. Psychophysiol. 38, 283–299. 10.1016/S0167-8760(00)00171-9 11102668

[B74] National Institute on Drug Abuse (2021). Is marijuana safe and effective as medicine? National Institute on drug abuse (NIDA). National institutes of health. Available at: https://nida.nih.gov/publications/research-reports/marijuana/marijuana-safe-effective-medicine (Accessed October 17, 2022).

[B75] Nennig S. E. Schank J. R. (2017). The role of NFkB in drug addiction: Beyond inflammation. Alcohol Alcohol. 52, 172–179. 10.1093/alcalc/agw098 28043969 PMC6410896

[B76] Nestby P. Vanderschuren L. J. M. J. de Vries T. J. Hogenboom F. Wardeh G. Mulder A. H. (1997). Ethanol, like psychostimulants and morphine, causes long-lasting hyperreactivity of dopamine and acetylcholine neurons of rat nucleus accumbens: Possible role in behavioural sensitization. Psychopharmacol. Berl. 133, 69–76. 10.1007/s002130050373 9335083

[B77] Nestler E. J. (2001). Molecular basis of long-term plasticity underlying addiction. Nat. Rev. Neurosci. 2, 119–128. 10.1038/35053570 11252991

[B78] Newton T. F. Cook I. A. Kalechstein A. D. Duran S. Monroy F. Ling W. (2003). Quantitative EEG abnormalities in recently abstinent methamphetamine dependent individuals. Clin. Neurophysiol. 114, 410–415. 10.1016/S1388-2457(02)00409-1 12705421

[B79] Nukitram J. Cheaha D. Kumarnsit E. (2021). Spectral power and theta-gamma coupling in the basolateral amygdala related with methamphetamine conditioned place preference in mice. Neurosci. Lett. 756, 135939. 10.1016/j.neulet.2021.135939 33945805

[B80] Ossato A. Uccelli L. Bilel S. Canazza I. di Domenico G. Pasquali M. (2017). Psychostimulant effect of the synthetic cannabinoid JWH-018 and AKB48: Behavioral, neurochemical, and dopamine transporter scan imaging studies in mice. Front. Psychiatry 8, 130. 10.3389/fpsyt.2017.00130 28824464 PMC5543288

[B81] Ota S. M. Suchecki D. Meerlo P. (2018). Chronic social defeat stress suppresses locomotor activity but does not affect the free-running circadian period of the activity rhythm in mice. Neurobiol. Sleep. Circadian Rhythms 5, 1–7. 10.1016/J.NBSCR.2018.03.002 31236507 PMC6584684

[B82] Ozaita A. Puighermanal E. Maldonado R. (2007). Regulation of PI3K/Akt/GSK-3 pathway by cannabinoids in the brain. J. Neurochem. 102, 1105–1114. 10.1111/j.1471-4159.2007.04642.x 17484726

[B83] Parikh V. Naughton S. X. Shi X. Kelley L. K. Yegla B. Tallarida C. S. (2014). Cocaine-induced neuroadaptations in the dorsal striatum: Glutamate dynamics and behavioral sensitization. Neurochem. Int. 75, 54–65. 10.1016/J.NEUINT.2014.05.016 24911954

[B84] Park H. Han K.-S. Seo J. Lee J. Dravid S. M. Woo J. (2015). Channel-mediated astrocytic glutamate modulates hippocampal synaptic plasticity by activating postsynaptic NMDA receptors. Mol. Brain 8, 7. 10.1186/s13041-015-0097-y 25645137 PMC4320468

[B85] Parsons L. H. Hurd Y. L. (2015). Endocannabinoid signalling in reward and addiction. Nat. Rev. Neurosci. 16, 579–594. 10.1038/nrn4004 26373473 PMC4652927

[B86] Paxinos G. Franklin K. B. J. (2001). Paxinos and franklin’s the mouse brain in stereotaxic coordinates. Available at: https://books.google.com/books?hl=en&lr=&id=x3aQDwAAQBAJ&oi=fnd&pg=PP1&dq=related:2wOjGlM4MNoJ:scholar.google.com/&ots=mAGLt_LNcu&sig=LmcHDk5xWmonT8ydLdNV2Lue5J0 (Accessed October 10, 2022).

[B87] Perdikaris P. Tsarouchi M. Fanarioti E. Natsaridis E. Mitsacos A. Giompres P. (2018). Long lasting effects of chronic WIN55,212-2 treatment on mesostriatal dopaminergic and cannabinoid systems in the rat brain. Neuropharmacology 129, 1–15. 10.1016/j.neuropharm.2017.11.005 29113897

[B88] Pintori N. Castelli M. P. Miliano C. Simola N. Fadda P. Fattore L. (2021). Repeated exposure to JWH-018 induces adaptive changes in the mesolimbic and mesocortical dopaminergic pathways, glial cells alterations, and behavioural correlates. Br. J. Pharmacol. 178, 3476–3497. 10.1111/bph.15494 33837969 PMC8457172

[B89] Polissidis A. Chouliara O. Galanopoulos A. Marselos M. Papadopoulou-Daifoti Z. Antoniou K. (2009). Behavioural and dopaminergic alterations induced by a low dose of WIN 55,212-2 in a conditioned place preference procedure. Life Sci. 85, 248–254. 10.1016/j.lfs.2009.05.015 19508876

[B90] Post R. M. Kalivas P. (2013). Bipolar disorder and substance misuse: Pathological and therapeutic implications of their comorbidity and cross-sensitisation. Br. J. Psychiatry 202, 172–176. 10.1192/BJP.BP.112.116855 23457180 PMC4340700

[B91] Qiu J. Yan Z. Tao K. Li Y. Li Y. Li J. (2016). Sinomenine activates astrocytic dopamine D2 receptors and alleviates neuroinflammatory injury via the CRYAB/STAT3 pathway after ischemic stroke in mice. J. Neuroinflammation 13, 263. 10.1186/s12974-016-0739-8 27724964 PMC5057372

[B92] Ramos K. M. Lewis M. T. Morgan K. N. Crysdale N. Y. Kroll J. L. Taylor F. R. (2010). Spinal upregulation of glutamate transporter GLT-1 by ceftriaxone: Therapeutic efficacy in a range of experimental nervous system disorders. Neuroscience 169, 1888–1900. 10.1016/J.NEUROSCIENCE.2010.06.014 20547213 PMC2918694

[B93] Reid M. S. Flammino F. Howard B. Nilsen D. Prichep L. S. (2006). Topographic imaging of quantitative EEG in response to smoked cocaine self-administration in humans. Neuropsychopharmacology 31, 872–884. 10.1038/sj.npp.1300888 16192989

[B94] Ren Z. Yang M. Guan Z. Yu W. (2018). Astrocytic α7 nicotinic receptor activation inhibits amyloid-β aggregation by upregulating endogenous αB-crystallin through the PI3K/akt signaling pathway. Curr. Alzheimer Res. 16, 39–48. 10.2174/1567205015666181022093359 30345917

[B95] Ribeiro Do Couto B. Aguilar M. A. Lluch J. Rodríguez-Arias M. Miñarro J. (2009). Social experiences affect reinstatement of cocaine-induced place preference in mice. Psychopharmacol. Berl. 207, 485–498. 10.1007/s00213-009-1678-1 19798482

[B96] Rodrigues L. C. M. Gobira P. H. de Oliveira A. C. Pelição R. Teixeira A. L. Moreira F. A. (2014). Neuroinflammation as a possible link between cannabinoids and addiction. Acta Neuropsychiatr. 26, 334–346. 10.1017/neu.2014.24 25455257

[B97] Rodríguez-Arias M. Roger-Sánchez C. Vilanova I. Revert N. Manzanedo C. Miñarro J. (2016). Effects of cannabinoid exposure during adolescence on the conditioned rewarding effects of WIN 55212-2 and cocaine in mice: Influence of the novelty-seeking trait. Neural Plast. 2016, 6481862. 10.1155/2016/6481862 26881125 PMC4736006

[B98] Rygula R. Abumaria N. Flügge G. Fuchs E. Rüther E. Havemann-Reinecke U. (2005). Anhedonia and motivational deficits in rats: Impact of chronic social stress. Behav. Brain Res. 162, 127–134. 10.1016/j.bbr.2005.03.009 15922073

[B99] Sales-Carbonell C. Rueda-Orozco P. E. Soria-Gómez E. Buzsáki G. Marsicano G. Robbe D. (2013). Striatal GABAergic and cortical glutamatergic neurons mediate contrasting effects of cannabinoids on cortical network synchrony. Proc. Natl. Acad. Sci. U. S. A. 110, 719–724. 10.1073/pnas.1217144110 23269835 PMC3545808

[B100] Sayson L. V. Custodio R. J. P. Ortiz D. M. Lee H. J. Kim M. Jeong Y. (2020). The potential rewarding and reinforcing effects of the substituted benzofurans 2-EAPB and 5-EAPB in rodents. Eur. J. Pharmacol. 885, 173527. 10.1016/j.ejphar.2020.173527 32871174

[B101] Schell M. T. Spitzer A. L. Johnson J. A. Lee D. Harris H. W. (2005). Heat shock inhibits NF-kB activation in a dose- and time-dependent manner. J. Surg. Res. 129, 90–93. 10.1016/j.jss.2005.05.025 16139305

[B102] Scherma M. Dessì C. Muntoni A. L. Lecca S. Satta V. Luchicchi A. (2016). Adolescent Δ 9-tetrahydrocannabinol exposure alters WIN55,212-2 self-administration in adult rats. Neuropsychopharmacology 41, 1416–1426. 10.1038/npp.2015.295 26388146 PMC4793126

[B103] Schmitz N. Richert L. (2020). Pharmacists and the future of cannabis medicine. J. Am. Pharm. Assoc. 60, 207–211. 10.1016/J.JAPH.2019.11.007 31870860

[B104] Shao W. Zhang S. Z. Tang M. Zhang X. H. Zhou Z. Yin Y. Q. (2013). Suppression of neuroinflammation by astrocytic dopamine D2 receptors via αb-crystallin. Nature 494, 90–94. 10.1038/nature11748 23242137

[B105] Skalisz L. L. Beijamini V. Joca S. L. Vital M. A. B. F. da Cunha C. Andreatini R. (2002). Evaluation of the face validity of reserpine administration as an animal model of depression-Parkinson’s disease association. Prog. Neuropsychopharmacol. Biol. Psychiatry 26, 879–883. 10.1016/S0278-5846(01)00333-5 12369260

[B106] Skosnik P. D. D’Souza D. C. Steinmetz A. B. Edwards C. R. Vollmer J. M. Hetrick W. P. (2012). The effect of chronic cannabinoids on broadband eeg neural oscillations in humans. Neuropsychopharmacology 37, 2184–2193. 10.1038/npp.2012.65 22713908 PMC3422484

[B107] Smaga I. Gawlińska K. Frankowska M. Wydra K. Sadakierska-Chudy A. Suder A. (2020). Extinction training after cocaine self-administration influences the epigenetic and genetic machinery responsible for glutamatergic transporter gene expression in male rat brain. Neuroscience 451, 99–110. 10.1016/J.NEUROSCIENCE.2020.10.001 33065231

[B108] Somade O. T. Ajayi B. O. Tajudeen N. O. Atunlute E. M. James A. S. Kehinde S. A. (2019). Camphor elicits up-regulation of hepatic and pulmonary pro-inflammatory cytokines and chemokines via activation of NF-kB in rats. Pathophysiology 26, 305–313. 10.1016/j.pathophys.2019.07.005 31377033

[B109] Sora I. Li B. Igari M. Hall F. S. Ikeda K. (2010). Transgenic mice in the study of drug addiction and the effects of psychostimulant drugs. Ann. N. Y. Acad. Sci. 1187, 218–246. 10.1111/J.1749-6632.2009.05276.X 20201856

[B110] Spanagel R. (2020). Cannabinoids and the endocannabinoid system in reward processing and addiction: From mechanisms to interventions. Dialogues Clin. Neurosci. 22, 241–250. 10.31887/DCNS.2020.22.3/RSPANAGEL 33162767 PMC7605022

[B111] Spanagel R. Sanchis-Segura C. (2003). The use of transgenic mice to study addictive behavior. Clin. Neurosci. Res. 3, 325–331. 10.1016/S1566-2772(03)00094-X

[B112] Sun L. Dong R. Xu X. Yang X. Peng M. (2017). Activation of cannabinoid receptor type 2 attenuates surgery-induced cognitive impairment in mice through anti-inflammatory activity. J. Neuroinflammation 14, 138. 10.1186/s12974-017-0913-7 28724382 PMC5518095

[B113] Sun Y. Li Y.-S. Yang J.-W. Yu J. Wu Y.-P. Li B.-X. (2014). Exposure to atrazine during gestation and lactation periods: Toxicity effects on dopaminergic neurons in offspring by downregulation of Nurr1 and VMAT2. Int. J. Mol. Sci. 15, 2811–2825. 10.3390/ijms15022811 24552878 PMC3958883

[B114] Sutton L. P. Caron M. G. (2015). Essential role of D1R in the regulation of mTOR complex1 signaling induced by cocaine. Neuropharmacology 99, 610–619. 10.1016/J.NEUROPHARM.2015.08.024 26314207 PMC4703076

[B115] Szumlinski K. K. Lominac K. D. Campbell R. R. Cohen M. Fultz E. K. Brown C. N. (2017). Methamphetamine addiction vulnerability: The glutamate, the bad, and the ugly. Biol. Psychiatry 81, 959–970. 10.1016/j.biopsych.2016.10.005 27890469 PMC5391296

[B116] Tai S. Fantegrossi W. E. (2014). Synthetic cannabinoids: Pharmacology, behavioral effects, and abuse potential. Curr. Addict. Rep. 1, 129–136. 10.1007/s40429-014-0014-y 26413452 PMC4582439

[B117] Tallon-Baudry C. Bertrand O. Hénaff M.-A. Isnard J. Fischer C. (2005). Attention modulates gamma-band oscillations differently in the human lateral occipital cortex and fusiform gyrus. Cereb. Cortex 15, 654–662. 10.1093/cercor/bhh167 15371290

[B118] Tampus R. Yoon S. S. de la Peña J. B. Botanas C. J. Kim H. J. Seo J. W. (2015). Assessment of the abuse liability of synthetic cannabinoid agonists JWH-030, JWH-175, and JWH-176. Biomol. Ther. Seoul. 23, 590–596. 10.4062/biomolther.2015.120 26535085 PMC4624076

[B119] Thomsen M. Caine S. B. (2011). False positive in the intravenous drug self-administration test in C57BL/6J mice. Behav. Pharmacol. 22, 239–247. 10.1097/FBP.0b013e328345f8f2 21522054 PMC3095106

[B120] Tsai S. J. (2007). Increased central brain-derived neurotrophic factor activity could be a risk factor for substance abuse: Implications for treatment. Med. Hypotheses 68, 410–414. 10.1016/J.MEHY.2006.05.035 16824691

[B121] Ucha M. Roura-Martínez D. Ambrosio E. Higuera-Matas A. (2020). The role of the mTOR pathway in models of drug-induced reward and the behavioural constituents of addiction. J. Psychopharmacol. 34, 1176–1199. 10.1177/0269881120944159 32854585

[B122] van de Giessen E. Weinstein J. J. Cassidy C. M. Haney M. Dong Z. Ghazzaoui R. (2017). Deficits in striatal dopamine release in cannabis dependence. Mol. Psychiatry 22, 68–75. 10.1038/mp.2016.21 27001613 PMC5033654

[B123] VanGuilder H. D. Vrana K. E. Freeman W. M. (2008). Twenty-five years of quantitative PCR for gene expression analysis. Biotechniques 44, 619–626. 10.2144/000112776 18474036

[B124] Vlachou S. Panagis G. (2014). Regulation of brain reward by the endocannabinoid system: A critical review of behavioral studies in animals. Curr. Pharm. Des. 20, 2072–2088. 10.2174/13816128113199990433 23829366

[B125] Volkow N. D. (2009). Substance use disorders in Schizophrenia - clinical implications of comorbidity. Schizophr. Bull. 35, 469–472. 10.1093/schbul/sbp016 19325163 PMC2669586

[B126] Vonder Haar C. Ferland J. M. N. Kaur S. Riparip L. K. Rosi S. Winstanley C. A. (2019). Cocaine self-administration is increased after frontal traumatic brain injury and associated with neuroinflammation. Eur. J. Neurosci. 50, 2134–2145. 10.1111/ejn.14123 30118561 PMC6517083

[B127] Wang S.-H. Hsiao P.-C. Yeh L.-L. Liu C.-M. Liu C.-C. Hwang T.-J. (2018). Polygenic risk for schizophrenia and neurocognitive performance in patients with schizophrenia. Genes. Brain Behav. 17, 49–55. 10.1111/gbb.12401 28719030

[B128] Weinstein A. M. Rosca P. Fattore L. London E. D. (2017). Synthetic cathinone and cannabinoid designer drugs pose a major risk for public health. Front. Psychiatry 8, 156. 10.3389/fpsyt.2017.00156 28878698 PMC5572353

[B129] Wilson J. M. Kalasinsky K. S. Levey A. I. Bergeron C. Reiber G. Anthony R. M. (1996). Striatal dopamine nerve terminal markers in human, chronic methamphetamine users. Nat. Med. 2 (6), 699–703. 10.1038/nm0696-699 8640565

[B130] Wood N. C. Hamilton I. Axon A. T. R. Khan S. A. Quirke P. Mindham R. H. S. (1987). Abnormal intestinal permeability. Br. J. Psychiatry 150, 853–856. 10.1192/bjp.150.6.853 3651740

[B131] Xi Z. X. Peng X. Q. Li X. Song R. Zhang H. Y. Liu Q. R. (2011). Brain cannabinoid CB- receptors modulate cocaine's actions in mice. Nat. Neurosci. 14, 1160–1166. 10.1038/nn.2874 21785434 PMC3164946

[B132] Xie S. Wang X. (2022). CRYAB reduces cigarette smoke-induced inflammation, apoptosis, and oxidative stress by retarding PI3K/Akt and NF-κB signaling pathways in human bronchial epithelial cells. Allergol. Immunopathol. Madr. 50, 23–29. 10.15586/AEI.V50I5.645 36086960

[B133] Xu F. Yu H. Liu J. Cheng L. (2013). αb-crystallin regulates oxidative stress-induced apoptosis in cardiac H9c2 cells via the PI3K/AKT pathway. Mol. Biol. Rep. 40, 2517–2526. 10.1007/s11033-012-2332-2 23212619

[B134] Xu H. Cheng C. L. Chen M. Manivannan A. Cabay L. Pertwee R. G. (2007). Anti-inflammatory property of the cannabinoid receptor-2-selective agonist JWH-133 in a rodent model of autoimmune uveoretinitis. J. Leukoc. Biol. 82, 532–541. 10.1189/JLB.0307159 17537989

[B135] Xu L. Nan J. Lan Y. (2020). The nucleus accumbens: A common target in the comorbidity of depression and addiction. Front. Neural Circuits 14, 37. 10.3389/fncir.2020.00037 32694984 PMC7338554

[B136] Xu W. Guo Y. Huang Z. Zhao H. Zhou M. Huang Y. (2019). Small heat shock protein CRYAB inhibits intestinal mucosal inflammatory responses and protects barrier integrity through suppressing IKKβ activity. Mucosal Immunol. 12, 1291–1303. 10.1038/s41385-019-0198-5 31481750

[B137] Yazdani N. Parker C. C. Shen Y. Reed E. R. Guido M. A. Kole L. A. (2015). Hnrnph1 is A quantitative trait gene for methamphetamine sensitivity. PLoS Genet. 11, e1005713. 10.1371/journal.pgen.1005713 26658939 PMC4675533

[B138] Young A. M. H. Campbell E. Lynch S. Suckling J. Powis S. J. (2011). Aberrant NF-kappaB expression in autism spectrum condition: A mechanism for neuroinflammation. Front. Psychiatry 2, 27. 10.3389/fpsyt.2011.00027 21629840 PMC3098713

[B139] Zanettini C. Scaglione A. Keighron J. D. Giancola J. B. Lin S.-C. Newman A. H. (2019). Pharmacological classification of centrally acting drugs using EEG in freely moving rats: An old tool to identify new atypical dopamine uptake inhibitors. Neuropharmacology 161, 107446. 10.1016/j.neuropharm.2018.11.034 30481526 PMC8369976

[B140] Zehra A. Burns J. Liu C. K. Manza P. Wiers C. E. Volkow N. D. (2018). Cannabis addiction and the brain: A review. J. Neuroimmune Pharmacol. 13, 438–452. 10.1007/s11481-018-9782-9 29556883 PMC6223748

[B141] Zhang J. F. Liu J. Wu J. L. Li W. F. Chen Z. W. Yang L. S. (2019). Progression of the role of CRYAB in signaling pathways and cancers. Onco Targets Ther. 12, 4129–4139. 10.2147/OTT.S201799 31239701 PMC6553995

[B142] Zhang Y. Crofton E. J. Li D. Lobo M. K. Fan X. Nestler E. J. (2014). Overexpression of DeltaFosB in nucleus accumbens mimics the protective addiction phenotype, but not the protective depression phenotype of environmental enrichment. Front. Behav. Neurosci. 8, 297. 10.3389/fnbeh.2014.00297 25221490 PMC4148937

[B143] Zhu R. Bu Q. Fu D. Shao X. Jiang L. Guo W. (2018). Toll-like receptor 3 modulates the behavioral effects of cocaine in mice. J. Neuroinflammation 15, 93–11. 10.1186/s12974-018-1130-8 29571298 PMC5865345

[B144] Zhu Z. Li R. Stricker R. Reiser G. (2015). Extracellular α-crystallin protects astrocytes from cell death through activation of MAPK, PI3K/Akt signaling pathway and blockade of ROS release from mitochondria. Brain Res. 1620, 17–28. 10.1016/j.brainres.2015.05.011 25998538

