# Supplementary material for: Deletion of Cryab increases the vulnerability of mice to the addiction-like effects of the cannabinoid JWH-018 via upregulation of striatal NF-κB expression
Source: Front Pharmacol. 2023 Mar 16;14:1135929. doi: 10.3389/fphar.2023.1135929 (PMC10060981; doi:10.3389/fphar.2023.1135929)
Supplement: Supplementary file 1 [file DataSheet2.pdf]

Supplementary Methods and Results

Deletion of *Cryab* increases the vulnerability of mice to the addiction-like effects of the cannabinoid JWH-018 via upregulation of striatal NF-κB expression

Leandro Val Sayson[1], Darlene Mae Ortiz[1], Hyun Jun Lee[1], Mikyung Kim[2], Raly James Perez Custodio[3], Jaesuk Yun[4], Chae Hyeon Lee[5], Yong Sup Lee[5], Hye Jin Cha[6], Jae Hoon Cheong[7*], and Hee Jin Kim[1*]

[*]Corresponding Authors:

Tel: +82-2-3399-1609, Fax: +82-2-3399-1617

E-mail address: cheongjh@jbnu.ac.kr (J.H. Cheong)

hjkim@syu.ac.kr (H.J. Kim)

1. Methods

1.1 Lippopolysaccharide (LPS) pre-treatment before conditioned place preference (CPP) test

The apparatus comprised two compartments (dimensions = 17.4 × 12.7 × 12.7 cm) with a removable guillotine door that separated the compartments. One compartment had smooth black walls and white flooring, whereas the other had white-dotted black walls and textured white flooring. An illumination of 12 lux was maintained throughout the experiment. A computer system (EthoVision) was used for recording animal movements and stay durations in the compartments. The protocol was performed according to previous studies with some modifications. Wild-type (WT) mice were administered with LPS (1 mg/kg body weight) or saline (SAL) for one day. They were allowed to recover for seven days before proceeding with the actual experiment. The test comprised the following three phases: (A) habituation (days 1–3) and pre-conditioning (day 4; 15 min), (B) conditioning (days 5–12; 30 min), and (C) post-conditioning (day 13; 15 min). During habituation, mice were allowed to freely explore the entire apparatus. An initial trial (pre-conditioning) was performed to determine the stay duration of each mouse in each of the compartments. Mice were assigned to groups based on the pre-conditioning phase such that their non-preferred side was designated as the drug-paired compartment. In the conditioning phase, mice were administered JWH-018 (0.3 mg/kg body weight) or vehicle (VEH) and placed in the drug-paired compartment. On alternate days, the mice received VEH and were confined to the VEH-paired compartment. During the post-conditioning phase, the mice were not treated and were allowed to explore both compartments (similar to the pre-conditioning phase). The CPP score was calculated as the difference in the time spent by the mice in their respective drug-paired compartments between the post-conditioning and pre-conditioning phases.

1.2 Protein extraction and Western blotting

The striatum (STR) of LPS- or SAL-pretreated mice used in the CPP test was isolated similarly as described in the previous section. Protein extraction was done according to previous methods with slight modifications. Brain tissues were lysed in 400 μL homogenization buffer (RIPA assay buffer [Biosesang Inc., Seongnam, Korea] supplemented with cOmplete™ ULTRA protease inhibitor cocktail tablets [05892791001, Sigma-Aldrich] and PhosSTOP™ phosphatase inhibitor cocktail tablets [04906845001, Sigma-Aldrich]). The

tissue extracts were centrifuged at 16000 *g* at 4 °C for 20 min. Western blotting protocol was done according to previous studies. The samples were then heated at 95 °C for 5 min. Protein lysates (20 μg) were subjected to sodium dodecyl sulfate-polyacrylamide gel electrophoresis on a 12% gel. The resolved proteins were transferred onto nitrocellulose membranes. The membrane was blocked with 5% bovine serum albumin (BSA) in prepared in Tris-buffered saline containing 0.1% Tween-20 (TBST) for 1 h and then incubated overnight with anti-mouse NF-κB (MBS9400350; MyBioSource, Inc.) antibody at 4 °C. Then, the membrane was washed with TBST and incubated with horseradish peroxidase-conjugated anti-mouse secondary antibody (1:5000) for 1 h. Protein bands were visualized based on enhanced chemiluminescence (Clarity Western ECL; Bio-Rad Laboratories, Hercules, CA, USA) using the ChemiDoc Imaging System (Image Lab software, version 6.0; Bio-Rad). The levels of phosphorylation-independent proteins were normalized to those of β-actin. Fold change was determined by normalizing the values of the test groups to those of the SAL+VEH groups.

1.4 Statistical analyses

All mice were randomized for treatment. The researchers were blinded to the treatment of the animals while conducting the tests and analyzing the data. Statistical analyses were performed using GraphPad Prism v7 (GraphPad Software Inc., San Diego, CA, USA). Data are presented as the mean ± standard error of the mean (S.E.M.). The means were analyzed using one-way analysis of variance (ANOVA), followed by Tukey's multiple comparison test. Differences were considered statistically significant at $p < 0.05$.

2. Results

2.1 LPS-pretreatment modestly enhanced JWH-018 CPP and upregulated NF-κB in mice

JWH-018-induced CPP score (Supplementary Figure 1A, $F_{3,24} = 3.11$; $p < 0.05$) and NF-κB expression (Supplementary Figure 1C, $F_{3,12} = 3.72$; $p < 0.05$) were different among treatment groups. LPS pre-treatment resulted in higher JWH-018-induced CPP score compared to VEH counterpart ($p < 0.05$) and higher NF-κB expression compared to SAL pre-treated counterpart ($p < 0.05$).

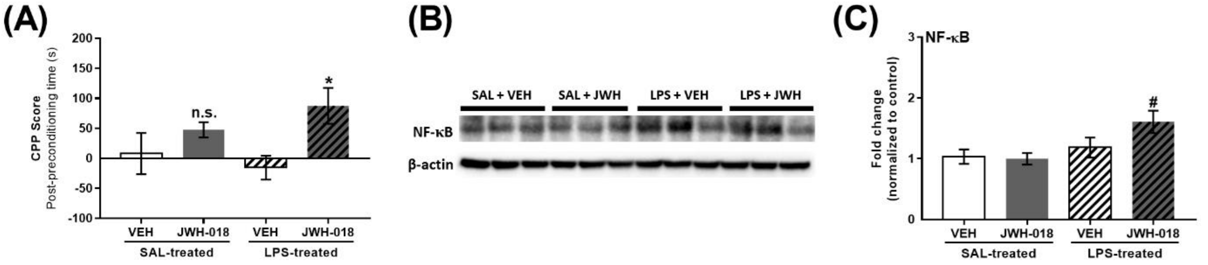

Supplementary Figure 1: Effect of lipopolysaccharide (LPS) on JWH-018 conditioned place preference (CPP) in wild-type (WT) mice. (A) CPP score of mice exposed to JWH-018 (0.3 mg/kg). $n = 7$. (B) Representative blots showing NF-κB expression in the striatum (STR) of mice. (C) NF-κB levels in LPS- or SAL-treated WT mice conditioned with JWH-018. Data expressed as mean ± S.E.M. $n = 4$. $^{*}p < 0.05$ (vs. VEH; Tukey post-hoc analysis). $^{\#}p < 0.05$ (vs. SAL-treated; Tukey post-hoc analysis).
