# Supplementary material for: Deletion of Cryab increases the vulnerability of mice to the addiction-like effects of the cannabinoid JWH-018 via upregulation of striatal NF-κB expression
Source: Front Pharmacol. 2023 Mar 16;14:1135929. doi: 10.3389/fphar.2023.1135929 (PMC10060981; doi:10.3389/fphar.2023.1135929)
Supplement: Supplementary file 2 [file Table1.DOCX]

| **Gene** | **Forward Sequence (5’ 🡪 3’)** | **Reverse Sequence (5’ 🡪 3’)** |
| --- | --- | --- |
| *Cnr1* | AAGTCGATCTTAGACGGCCTT | TCCTAATTTGGATGCCATGTCTC |
| *Cnr2* | ACGGTGGCTTGGAGTTCAAC | GCCGGGAGGACAGGATAAT |
| *Faah* | GTATCGCCAGTCCGTCATTG | GCCTATACCCTTTTTCATGCCC |
| *Mgll* | ACCATGCTGTGATGCTCTCTG | CAAACGCCTCGGGGATAACC |
| *Dat* | AAATGCTCCGTGGGACCAATG | GTCTCCCGCTCTTGAACCTC |
| *Drd1* | ATGGCTCCTAACACTTCTACCA | GGGTATTCCCTAAGAGAGTGGAC |
| *Drd2* | ACCTGTCCTGGTACGATGATG | GCATGGCATAGTAGTTGTAGTGG |
| *Vmat2* | ATGCTGCTCACCGTCGTAG | GGACAGTCGTGTTGGTCACAG |
| *Eaat2* | ACAATATGCCCAAGCAGGTAGA | CTTTGGCTCATCGGAGCTGA |
| *Eaat3* | CTTCCTACGGAATCACTGGCT | CGATCAGCGGCAAAATGACC |
| *Eaat4* | AGCAGCCACGGCAATAGTC | ATGCCAAGCTGACACCAATGA |
| *Gapdh* | AGGTCGGTGTGAACGGATTTG | TGTAGACCATGTAGTTGAGGTCA |

**Supplementary Table 1:** Sequences for oligonucleotides
