# Supplementary material for: Deletion of Cryab increases the vulnerability of mice to the addiction-like effects of the cannabinoid JWH-018 via upregulation of striatal NF-κB expression
Source: Front Pharmacol. 2023 Mar 16;14:1135929. doi: 10.3389/fphar.2023.1135929 (PMC10060981; doi:10.3389/fphar.2023.1135929)
Supplement: Supplementary file 3 [file Table2.docx]

**Supplementary Table 2:** Primary antibody information

| **Target** | **Catalog Number (Brand)** | **Host** |
| --- | --- | --- |
| CRYAB | MBS805894 (MyBioSource, Inc.) | Rabbit |
| PI3K (110α) | MBS2554577 (MyBioSource, Inc.) | Rabbit |
| *p*-AKT (Ser473) | MBS3016042 (MyBioSource, Inc.) | Rabbit |
| pan-AKT | MA5-14916 (Invitrogen) | Rabbit |
| GSK-3β (27C10) | 9315 (Cell Signaling Technology) | Rabbit |
| *p*-GSK-3β (Ser9) | 9336 (Cell Signaling Technology) | Rabbit |
| NF-κB (p65) | MBS9400350 (MyBioSource, Inc.) | Mouse |
| GFAP | LS-B15993 (LS Bio) | Rabbit |
| TNF-α | AMC3012 (Thermofisher) | Rabbit |
| IL-1β | MBS3000115 ( MyBioSource, Inc.) | Rabbit |
| IL-6 | SC28343 (Santa Cruz Biotechnology) | Mouse |
| GluA1 | 13185 (Cell Signaling Technology) | Rabbit |
| GluA2 | 13607 (Cell Signaling Technology) | Rabbit |
| *p*-CREB (Ser133) | 9198 (Cell Signaling Technology) | Rabbit |
| CREB | 9197 (Cell Signaling Technology) | Rabbit |
| ΔFosB | ab184938 (Abcam) | Rabbit |
| *p*-mTOR | 5536 (Cell Signaling Technology) | Rabbit |
| mTOR | 2972 (Cell Signaling Technology) | Rabbit |
| BDNF | ab108319 (Abcam) | Rabbit |
| β-actin | A5441 (Sigma-Aldrich) | Mouse |
