# Supplementary material for: Deletion of Cryab increases the vulnerability of mice to the addiction-like effects of the cannabinoid JWH-018 via upregulation of striatal NF-κB expression
Source: Front Pharmacol. 2023 Mar 16;14:1135929. doi: 10.3389/fphar.2023.1135929 (PMC10060981; doi:10.3389/fphar.2023.1135929)

CRYAB

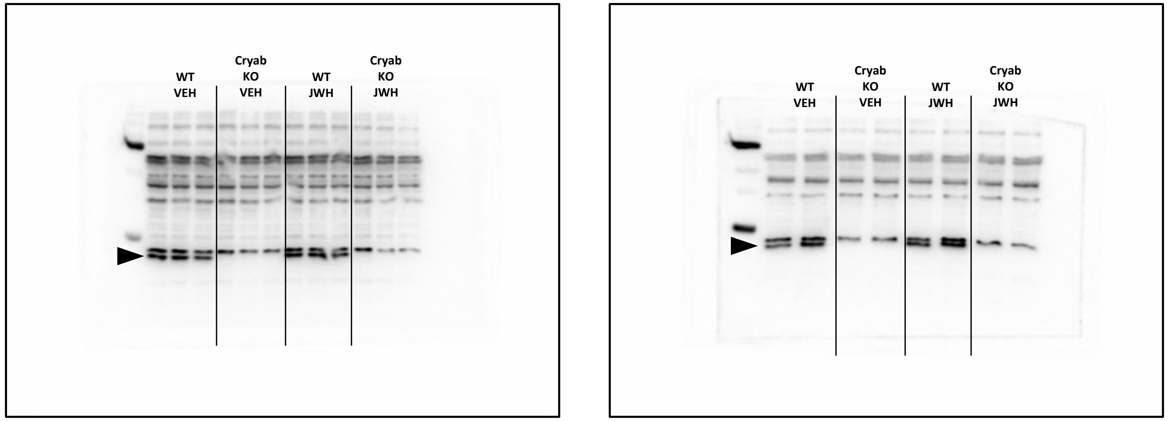

PI3K

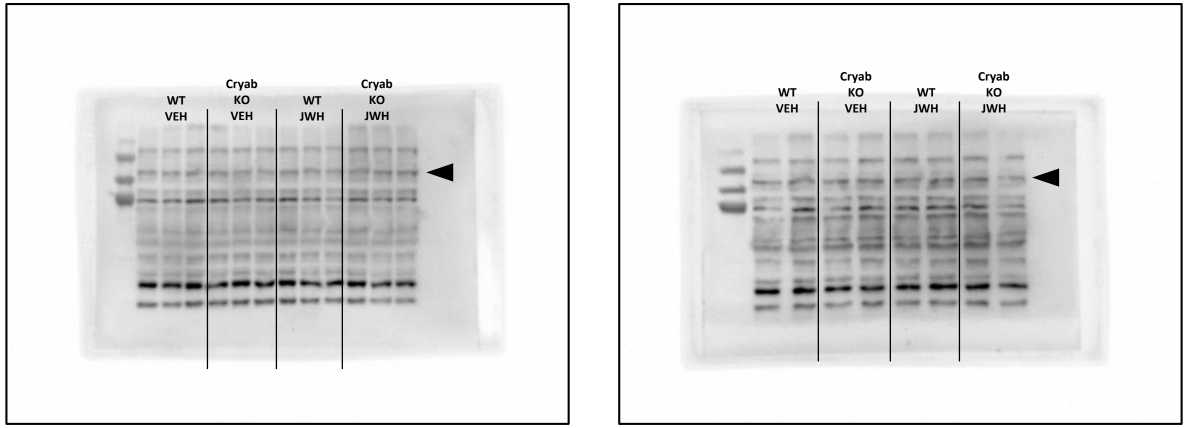

p-AKT

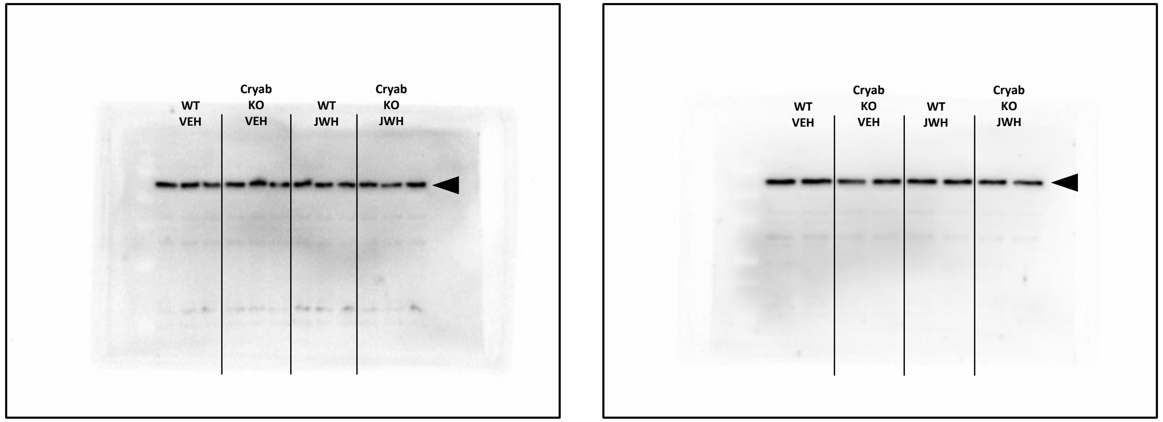

AKT

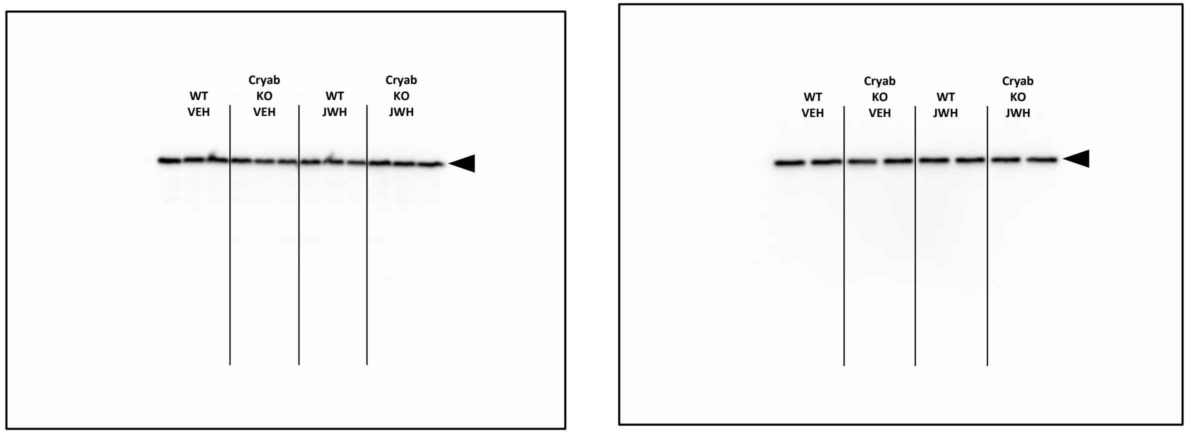

p-GSK3β

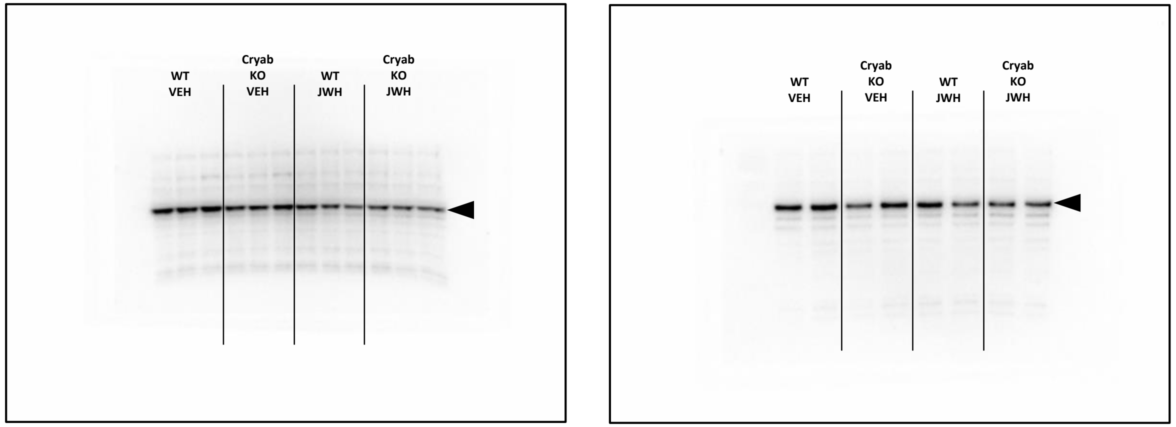

GSK3β

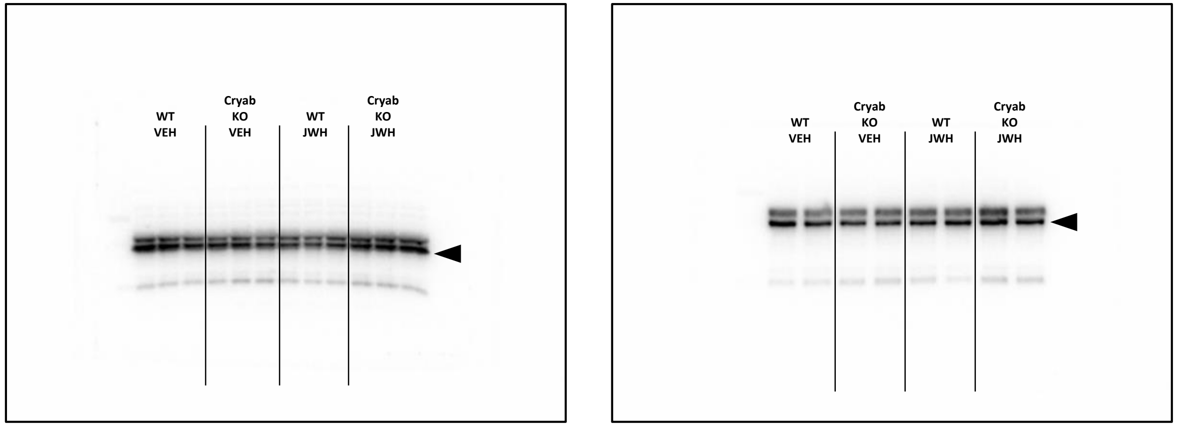

NF-κB

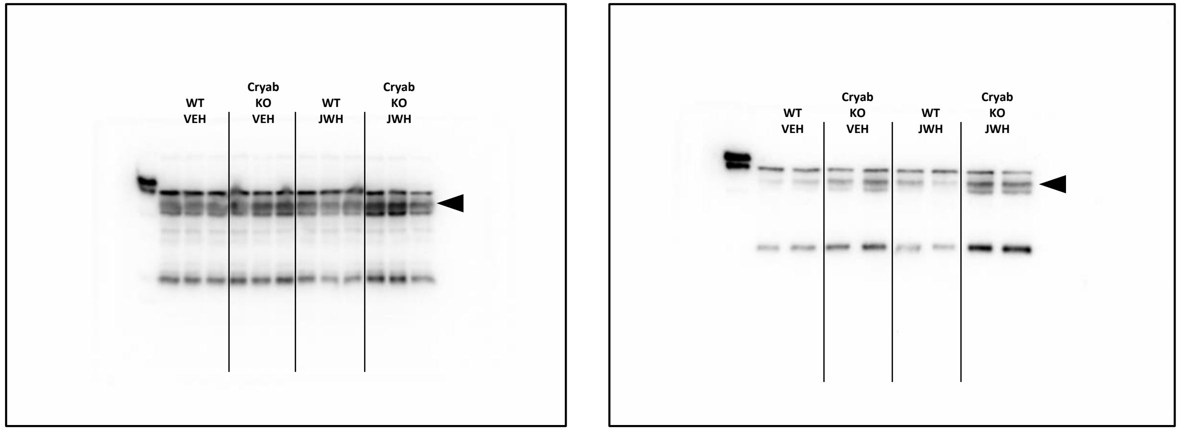

GFAP

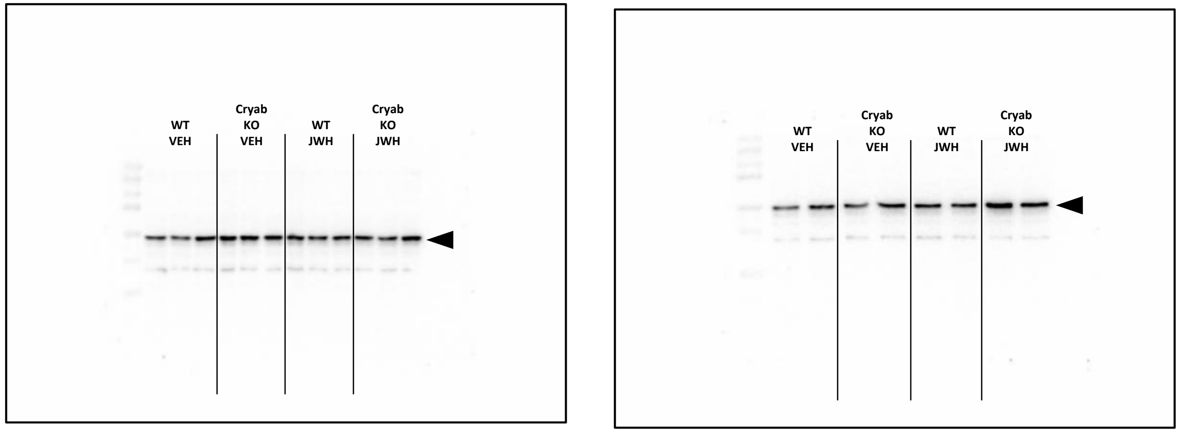

TNF-α

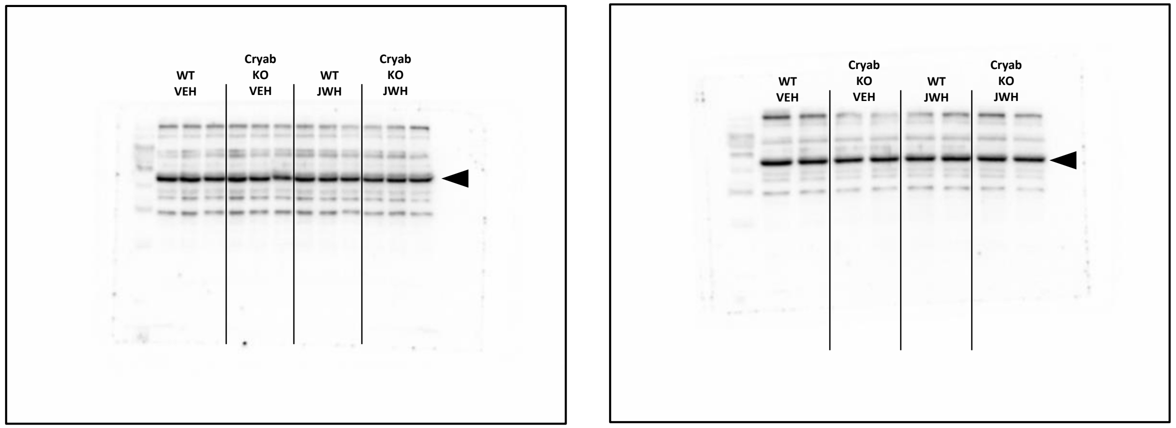

IL-1β

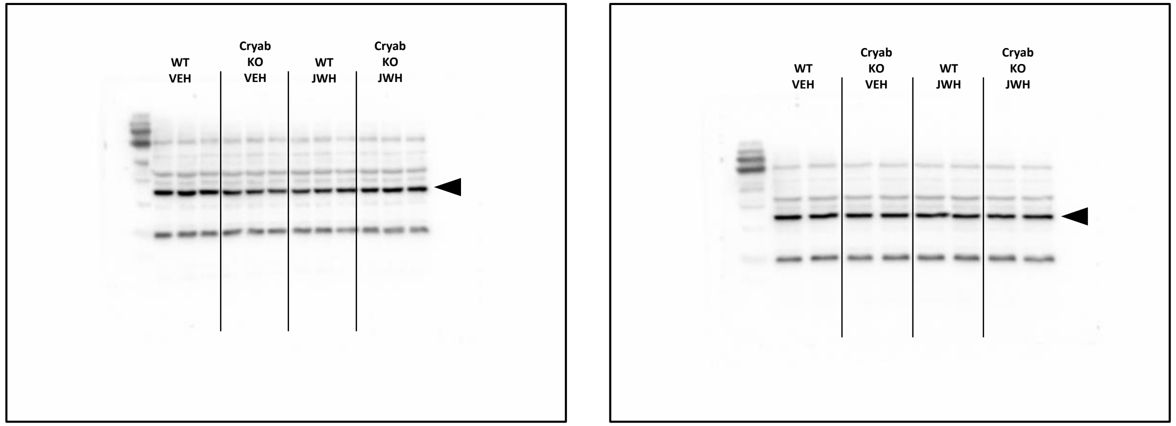

IL-6

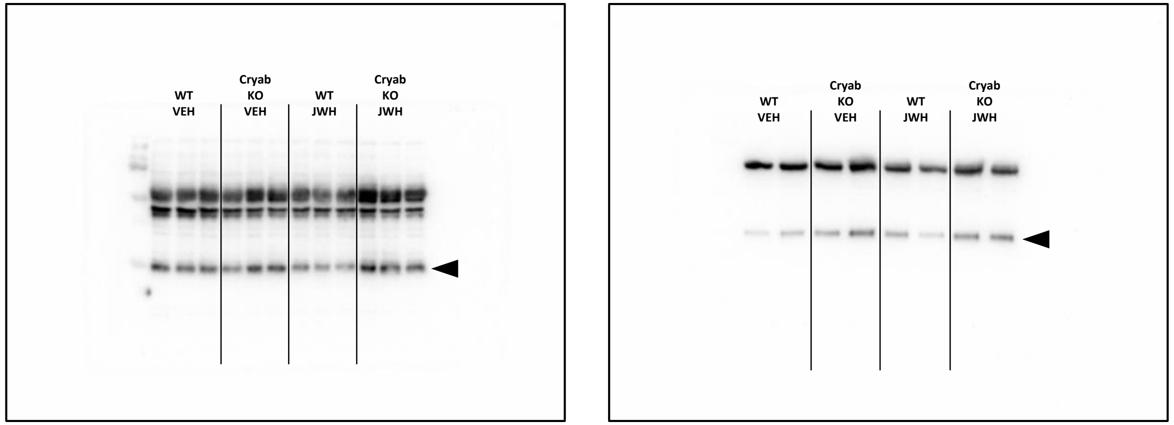

GluA1

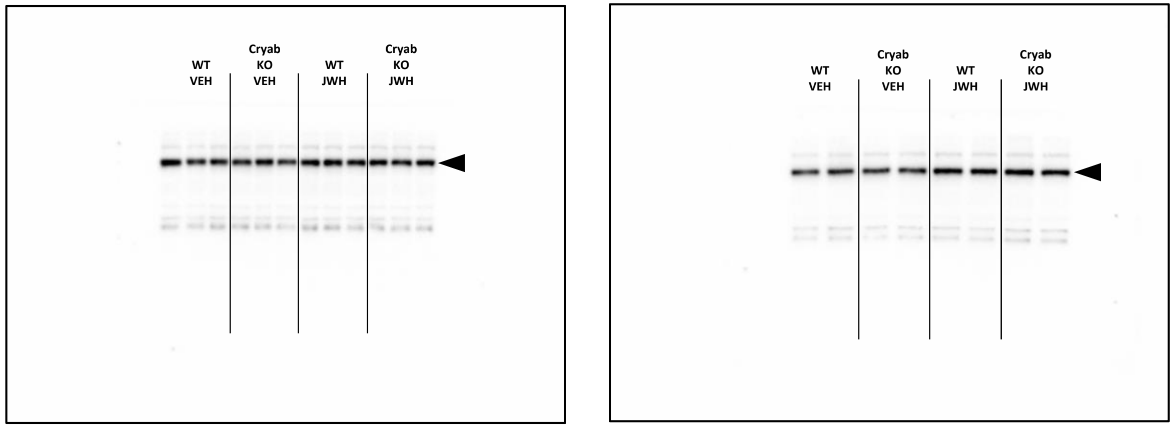

GluA2

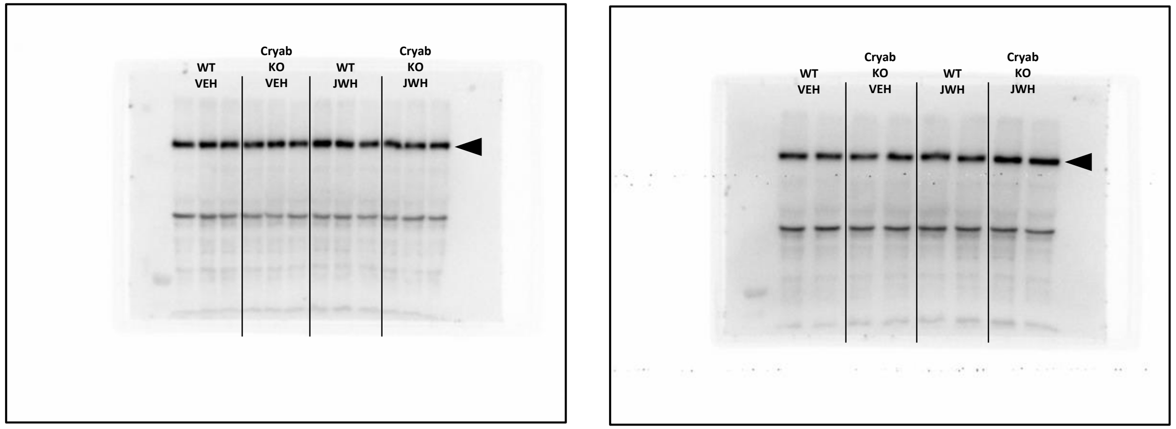

p-CREB

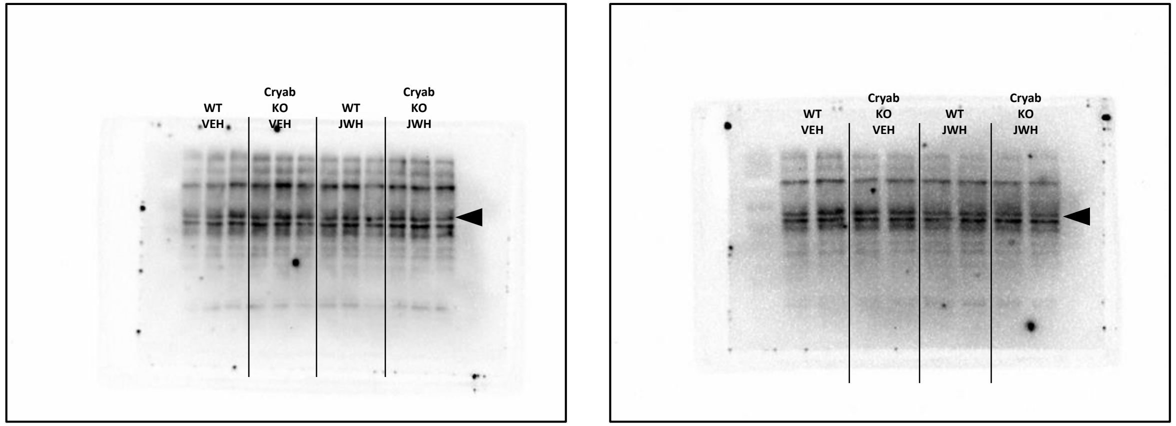

CREB

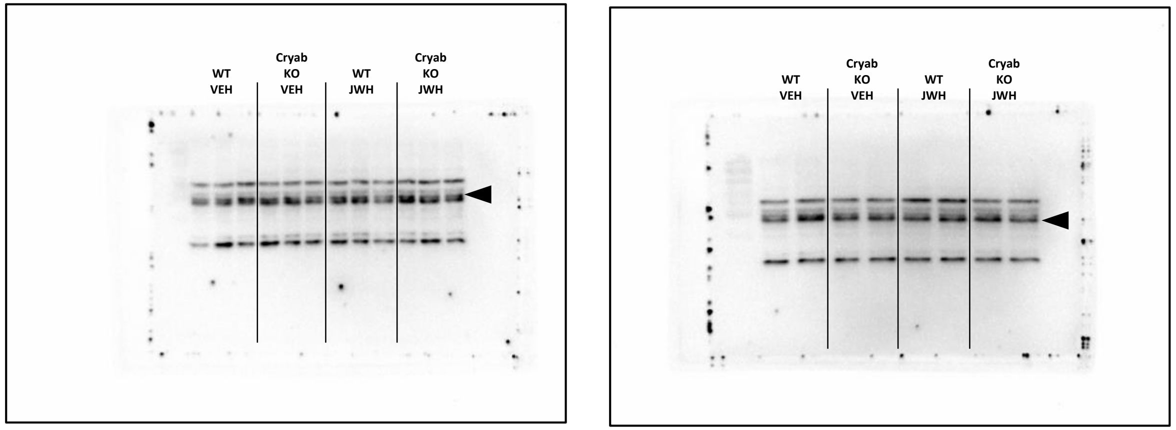

ΔFosB

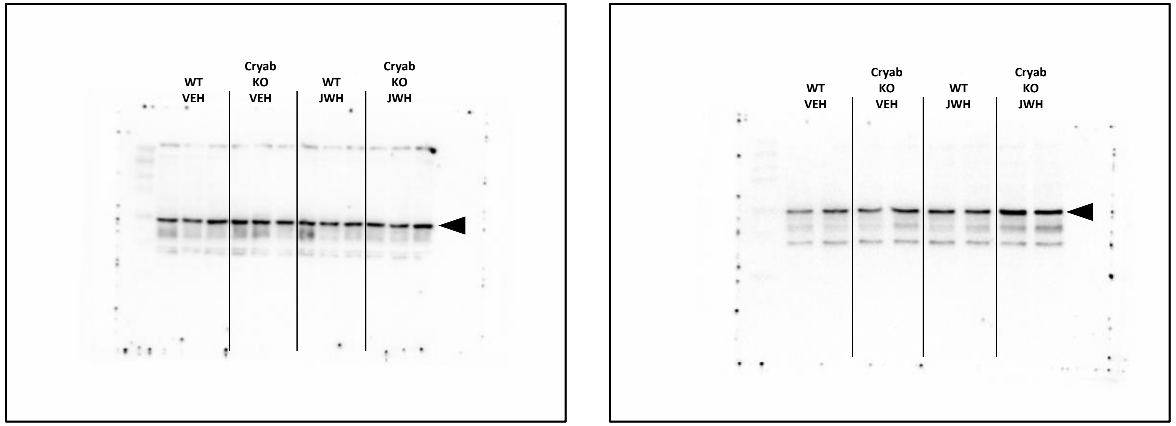

p-mTOR

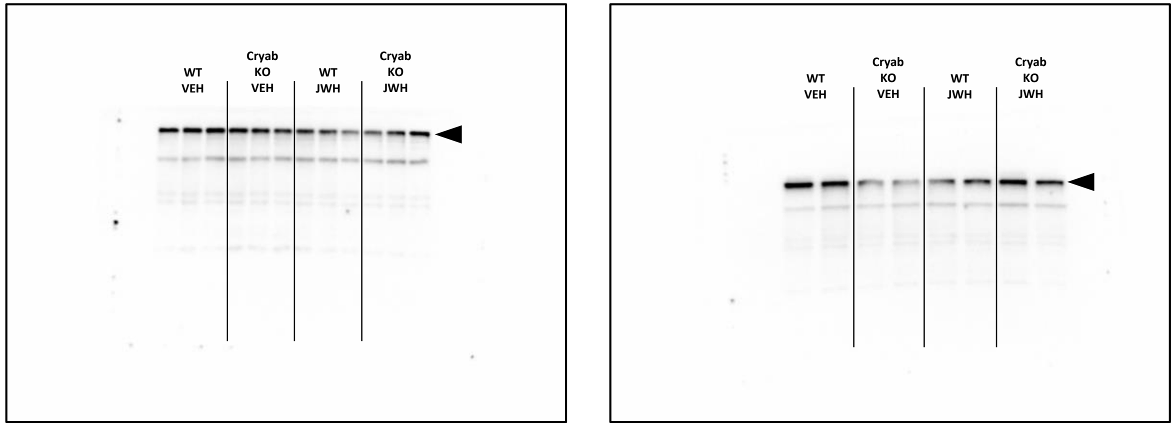

mTOR

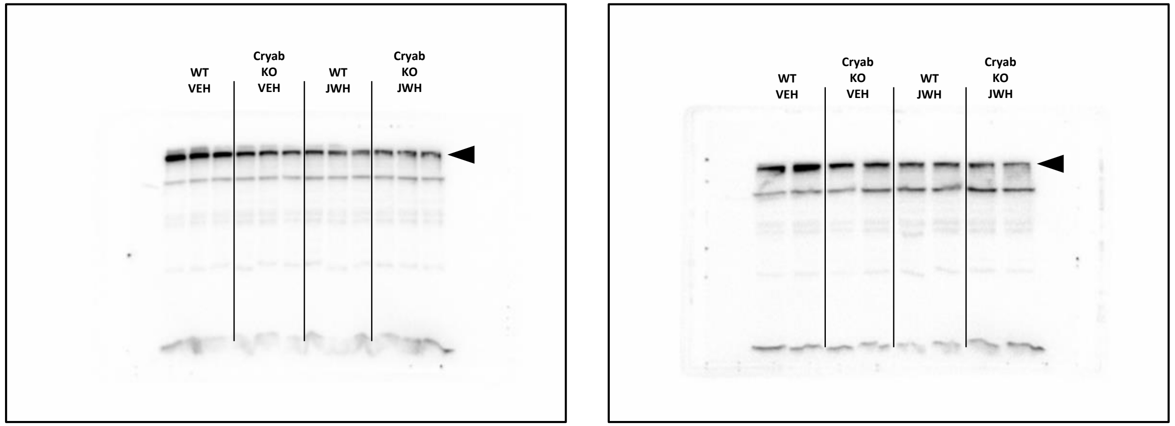

BDNF

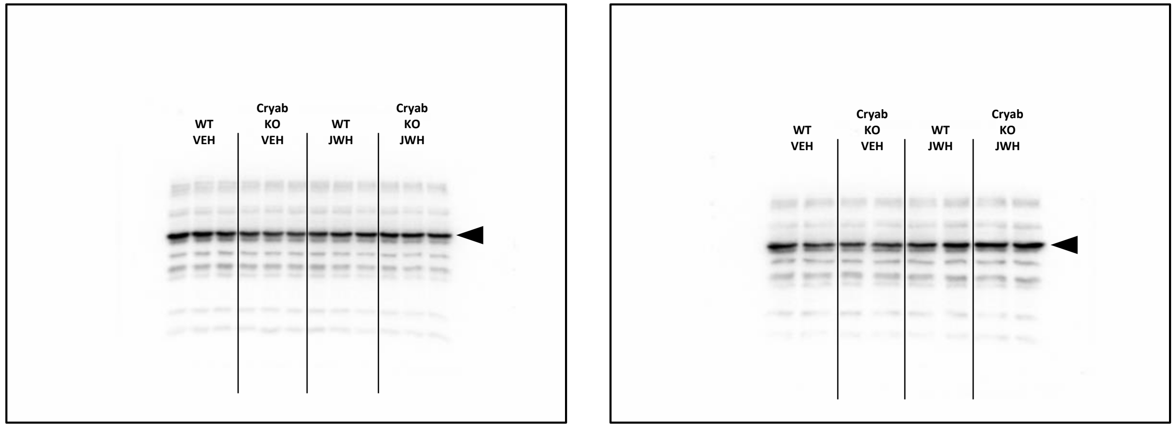

β-actin

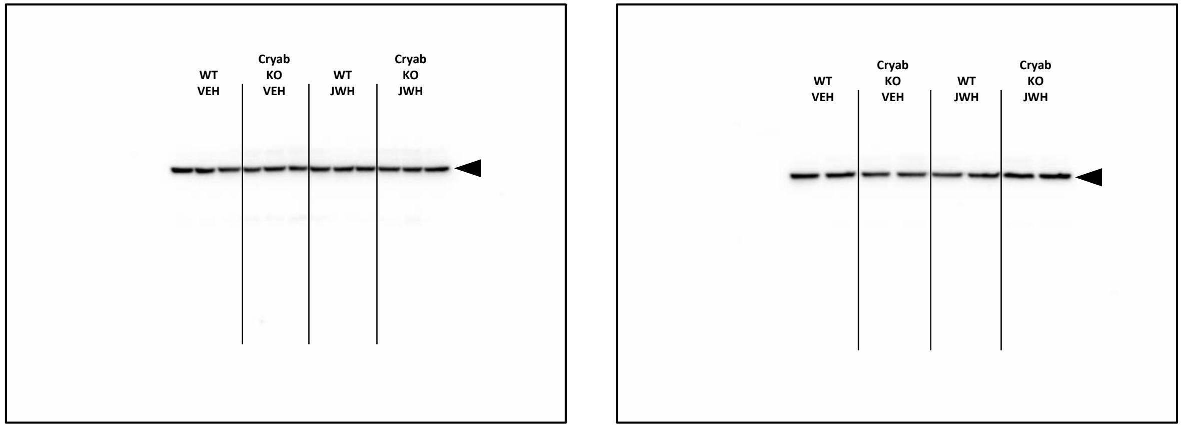

Supplement: Supplementary file 4 [file DataSheet1.PDF]
